# *Passiflora edulis* f. *flavicarpa* Extract Prevents Muscle Atrophy and Insulin Resistance in High‐Fat Diet–Induced Obese Rats via Regulating the Nrf2, NF‐κB, and IRS‐1/PI3K/AKT Signaling Pathways

**DOI:** 10.1155/omcl/5709962

**Published:** 2026-05-04

**Authors:** Nrarat Chobsuay, Pennapa Chonpathompikunlert, Jukkarin Srivilai, Wachirawadee Malakul, Nanteetip Limpeanchob, Sathid Aimjongjun, Sakara Tunsophon

**Affiliations:** ^1^ Department of Physiology, Faculty of Medical Science, Naresuan University, Phitsanulok, 65000, Thailand, nu.ac.th; ^2^ Biodiversity Research Center (BRC), Research and Development Group for Bio-Industries, Thailand Institute of Scientific and Technological Research (TISTR), Pathum Thani, 12120, Thailand, tistr.or.th; ^3^ Research and Innovation Center in Cosmetic Sciences and Natural Products, Department of Cosmetic Sciences, School of Pharmaceutical Sciences, University of Phayao, Phayao, 56000, Thailand, up.ac.th; ^4^ Unit of Excellence for Sustainable Innovation in Cosmetics, Natural Products and Pharmaceuticals (SICNP), School of Pharmaceutical Sciences, University of Phayao, Phayao, 56000, Thailand, up.ac.th; ^5^ Center of Excellence in Medical Biotechnology, Naresuan University, Phitsanulok, 65000, Thailand, nu.ac.th; ^6^ Department of Pharmacy Practice, Faculty of Pharmaceutical Sciences, Naresuan University, Phitsanulok, 65000, Thailand, nu.ac.th; ^7^ Center of Excellence for Innovation in Chemistry, Brain and Body Health Research Cluster, Naresuan University, Phitsanulok, 65000, Thailand, nu.ac.th; ^8^ Department of Biochemistry, Faculty of Medical Science, Naresuan University, Phitsanulok, 65000, Thailand, nu.ac.th

**Keywords:** high-fat diet, insulin resistance, muscle atrophy, obesity, *Passiflora edulis* f. *flavicarpa*

## Abstract

High‐fat diets (HFDs) are a key contributor to obesity and promote oxidative stress and inflammation, which are associated with muscle atrophy and insulin resistance (IR). *Passiflora edulis* exhibits anti‐obesity, antioxidant, and anti‐inflammatory effects. The study aimed to investigate the potential benefit of *P. edulis* f. *flavicarpa* (PF) extract in preventing obesity‐associated muscle atrophy and IR. The PF extract effectively inhibited cholesterol micelle solubility with an IC_50_ of 3431 µg/mL and decreased fat accumulation in 3T3‐L1 adipocytes. Furthermore, this study investigated a model of HFD–induced IR and muscle atrophy in rats. Thirty‐five male Sprague‐Dawley (SD) rats were induced with obesity by HFD and were administered 250 and 500 mg/kg/day of PF extract. Rats fed with an HFD were associated with fat accumulation and oxidative stress, which promoted inflammation, muscle damage, muscle atrophy, and IR in obese rats. However, administration of PF extract effectively mitigated these effects. The PF extract decreased fat accumulation in white adipose tissues and gastrocnemius (GAS) muscle by inhibiting fat absorption and synthesis, particularly *Cd36* and *Hmgcr*. The PF extract also notably reduced oxidative stress‐induced muscle inflammation and damage via elevating nuclear factor erythroid 2‐related factor 2 (Nrf2) and reducing nuclear factor kappa B (NF‐κB) expressions. Additionally, PF extract was found to mechanistically prevent muscle atrophy by inhibiting *Fbxo32*, *Trim63*, and B‐cell lymphoma 2 (BCL2)‐associated X (*Bax*) expressions, while enhancing *Bcl2* expression. We also found that PF extract mitigated muscle IR by upregulation of the insulin receptor substrate‐1/phosphatidylinositol‐3 kinase/protein kinase B (IRS‐1/PI3K/AKT) pathway and *Slc2a4* expression. The findings indicate that PF extract can prevent skeletal muscle loss and IR in obesity by modulating oxidative stress, inflammation, and activating IRS‐1/PI3K/AKT signaling pathway.

## 1. Introduction

Muscle atrophy is defined as a decline in muscle mass and impairment of muscular function (i.e., strength or performance). Diets, systemic diseases, or physical conditions including high‐fat, high‐sugar diets, aging‐related sarcopenia, diabetes, or obesity can all contribute to skeletal muscle atrophy [1]. Obesity can impair skeletal muscle function by reducing muscle mass at rates ranging from 75% to 92%, resulting in decreased mobility in obese people. Obesity is often associated with chronic pro‐inflammatory state and oxidative stress, both of which affect skeletal muscle function through disrupting the balance between protein synthesis and breakdown [2]. Oxidative stress accelerates muscle atrophy and results in muscle damage. This can lead to the probability of falling, decreased quality of life, and an increase in mortality [3]. Therefore, this condition is an important problem relating to public health as it could have serious consequences for people, particularly when aging. Prevention is crucial for current healthcare, as it helps minimize complications and further significant health issues while also keeping healthcare expenses low. Recently, diets high in saturated fats, particularly triglycerides (TGs), have been associated with an increase in obesity and other chronic disorders. Moreover, total cholesterol (TC) is also found in diets and is absorbed approximately 50% [4]. Thus, limiting fat absorption is one of strategies in individuals with obesity, hyperlipidemia, and type 2 diabetes [5]. Besides, high‐fat diet (HFD) causes a spike in plasma‐free fatty acids (FFAs) levels, which are transported into adipose tissue and muscle by fatty acid transporters and deposited as TGs. FFAs are then participated in various metabolic pathways to generate adenosine triphosphate (ATP) and release reactive oxygen species (ROS) as a by‐product [6, 7]. When ROS generation increases and exceeds the antioxidant enzyme capacity of defense systems, ROS can cause oxidative stress and damages various cellular components, including muscle tissue [8]. ROS activates transcription factors such as nuclear factor kappa B (NF‐κB), leading to the generation of pro‐inflammatory cytokines including tumor necrosis factor alpha (TNF‐α) and interleukin‐6 (IL‐6). However, our bodies have a defensive mechanism against oxidative stress and inflammation that activates nuclear factor erythroid 2‐related factor 2 (Nrf2) to elevate enzymatic antioxidants including superoxide dismutase (SOD), catalase (CAT), and glutathione peroxidase (GPx), which are the primary antioxidant systems [9]. Furthermore, ROS promoted skeletal muscle atrophy by increasing muscle‐specific RING finger protein 1 (TRIM63, also named MuRF1) expression [10, 11].

Previous research demonstrated that HFD causes insulin resistance (IR) via inhibiting the insulin receptor substrate‐1 (IRS‐1)/phosphatidylinositol‐3 kinase (PI3K)/protein kinase B (AKT) signaling pathway, which reduces the mammalian target of rapamycin (MTOR) activity while increasing the forkhead box O (FOXO) activity, resulting in muscular atrophy [12, 13]. Another mechanism implicated in HFD–induced muscle atrophy is mitochondrial apoptosis, as indicated by the BCL2‐associated X (BAX)/B‐cell lymphoma 2 (BCL2) ratio [14]. ROS and NF‐κB may activate BAX, which is important for controlling apoptotic cell death. Another apoptosis regulator is BCL2, which suppresses the actions of BAX and caspase‐3. However, ROS can reduce BCL2 expression and enhance cell death. Therefore, Nrf2 is important because it is the key regulator to modulate redox balance and inflammatory responses associated with muscle atrophy and IR. Furthermore, HFD enhances lipid accumulation, such as TG and TC, in skeletal muscle through increased fatty acid synthase (FAS) and 3‐hydroxy‐3‐methylglutaryl coenzyme A reductase (HMGCR) expressions, respectively [15]. Ectopic adipose infiltration in skeletal muscles, also known as myosteatosis, may lead to decreased muscular function and strength, IR, as well as muscle atrophy [16]. Simvastatin, an HMGCR inhibitor, is the most widely used as lipid‐lowering agent. Its mechanism is involved in de novo cholesterol biosynthesis. Although simvastatin is generally considered safe for clinical use, some studies have reported adverse effects, with inconsistent results among studies. A prior study found that simvastatin dosages of 40 and 80 mg daily resulted in higher alanine aminotransferase (ALT) levels, which led to hepatotoxicity [17]. Moreover, simvastatin is linked to muscle side effects such as muscle weight loss via regulating the expression of muscle atrophy genes such as muscle atrophy F‐box (Fbxo32, also known as Atrogin‐1), Trim63, and Myostatin (Mstn) [18–20]. Thus, to mitigate the potential risk of simvastatin‐induced muscle atrophy, we looked for a natural product that may prevent fat accumulation and ameliorate oxidative stress‐induced muscle loss in obesity.

*Passiflora edulis* (*P. edulis*), commonly known as passion fruit, contains a variety of components, including polyphenols, triterpenes, glycosides, carotenoids, cyanogenic glycosides, and others [21]. *Passiflora edulis* f. *flavicarpa* (PF) pulp has more quercetin and antioxidant capacity than the other types [22]. Additionally, gallic acid and caffeic acid are the major phenolic components found in passion fruit pulp extract [23]. Previous studies found that PF suppressed inflammation while also decreased oxidative stress and lipid profiles in the serum such as low‐density lipoprotein (LDL) [24, 25]. In addition, the previous study of *P. edulis* extract has demonstrated that it helps alleviate oxidative stress, inflammation, and IR in rats with hepatic steatosis induced by HFD [26]. However, the beneficial effects of PF extract on muscle atrophy require further study. Accordingly, the purpose of this study was to assess the preventive effects of PF extract on muscle atrophy and IR related to obesity in obese rats fed an HFD. We hypothesized that PF extract could protect against HFD‐induced skeletal muscle atrophy and IR in obese rats.

## 2. Methods

### 2.1. Preparation of PF Extract

The process of PF extraction and active compositions were previously reported by Sukketsiri et al. [27]. In brief, PF juice and pulp were blended, filtered, and freeze‐dried with 12.12% yield (*w*/*w*). The PF extract contained β‐carotene (191.7 µg/g extract) and γ‐tocopherol (20.03 µg/g extract). The total phenolic content (19.90 mg gallic acid equivalent/g extract), total flavonoid content (4.96 mg quercetin equivalent/g extract), total carotenoids content (1.23 mg β−carotene/g extract), and radical scavenging effects with approximately 56% were found in the PF extract. Two doses of 250 and 500 mg/kg body weight (BW) of PF extract were dissolved with 1 mL of distilled water for daily oral gavage to rats [28].

### 2.2. High‐Performance Liquid Chromatography (HPLC) Analysis

The Shimadzu Prominence UFLC system, equipped with an LC‐20AD pump and SPD‐20A 230 V UV‐Vis detector, was utilized to determine gallic acid and caffeic acid, Sigma–Aldrich (St. Louis, MO, USA), in the extract. Chromatographic separation was performed using a Phenomenex C‐18(2) column (250 mm × 4.6 mm, 5 µm particle size). The mobile phase A comprised of 1% (*v*/*v*) formic acid in deionized water, whereas 1% (*v*/*v*) formic acid in acetonitrile was used in the mobile Phase B. The mobile Phases A and B utilized gradient elution at different concentrations, as follows: the gradient declined from 90% to 85% in 0–6 min, then to 75% between 6 and 9 min, and finally at 75% until 16 min. The injection volume was 20 µL at a steady flow rate of 1.0 mL/min. The detection wavelengths for gallic acid and caffeic acid were set at 270 and 325 nm, respectively. The concentrations at 1, 10, 20, 30, 40, and 50 µg/mL of gallic acid and caffeic acid were prepared in deionized water and diluted as a standard stock solution. Then, the calibration curves were constructed by plotting peak area against concentration, and correlation coefficients (*R* ^2^) were determined. The PF extract was prepared at a concentration of 100 mg/mL in deionized water and filtered through a 0.45 µm membrane filter. The content of gallic acid and caffeic acid in the extract was measured and presented as micrograms of bioactive compound per gram of extract (µg/g).

### 2.3. Cholesterol Micellar Solubility

The cholesterol micellar solubility preparation method is based on Duangjai et al. [29]. In brief, the micellar solution was prepared with 0.6 mM phosphatidylcholine, 1 mM sodium taurocholate, and 10 mM cholesterol. A micellar solution was added to a tube containing PF extract (0.25, 0.5, 1, and 5 mg/mL). After that, the solution was mixed and incubated at 37°C for 3 h. The solution was centrifuged for 3 min before filtering through a 0.22 µm membrane. In the final step, a commercial assay kit (HUMAN Gesellschaft fur Biochemica und Diagnostics mbH, Wiesbaden, Germany) was used to measure the content of cholesterol in the solution. Data showed micellar cholesterol solubility, and the following formula was used to determine the inhibition of cholesterol micellar solubility: the percentage of inhibition of cholesterol solubility is 100 − ((cholesterol contents (treated) − (cholesterol contents (control)) × 100).

### 2.4. Cell Culture

3T3‐L1 cells were cultured according to a prior study [30]. 3T3‐L1 cells were cultured in Dulbecco’s modified eagle medium (DMEM) containing 10% bovine serum (BS) until confluent. On day 0, cells were cultured in DMEM media with BS, insulin, dexamethasone, and 3‐isobutyl‐1‐methylxanthine. After 78 h, the medium was changed and incubated for 7 days. 3T3‐L1 cell culture was maintained in an incubator with 5% CO_2_ and 90% humidity at 37°C.

### 2.5. Cell Viability

The MTT assay was performed according to the methodology provided by Reddy and Manjappara [31]. Preadipocyte 3T3‐L1 cells were seeded into 96‐well plates at a density of 3 × 10^3^ cells per well. Following that, the PF extract with concentrations of 5, 10, 50, 100, 500, and 1000 μg/mL were applied to the wells and incubated for 24, 48, and 72 h. After that, 20 μL of 3‐(4,5‐dimethylthiazol‐2‐yl)−2,5 diphenyl tetrazolium bromide (MTT) solution was added to each well and incubated at 37°C in a CO_2_ incubator. The solution was discarded and replaced with DMSO in each well, and then the absorbance was measured at 540 nm. The percentage of cell viability was determined by comparing to the control.

### 2.6. Oil Red O Staining

Oil red O staining and quantification were performed according to Mu et al. [30]. Cells in a 96‐well plate were rinsed with phosphate‐buffered saline (PBS) and fixed with 4% formaldehyde for 30 min. After washing with isopropanol, cells were incubated for 30 min with 0.5% Oil red O stain dissolved in isopropanol. Then, oil red O was washed out with PBS and dissolved in isopropanol. After that, absorbance was measured at 510 nm.

### 2.7. Animal Studies

The experiments followed ARRIVE guidelines and relevant regulations [32]. Seven‐week‐old male Sprague‐Dawley (SD) rats weighing between 150‐200 g were placed in cages with regulated temperature (22°C) and humidity (55% ± 10%) and were subjected to a 12‐h light and 12‐h dark cycle. Throughout a week‐long acclimatization period, they were granted unrestricted access to food and water. Following acclimatization, the rats were randomly distributed into five groups, each consisting of seven rats. These groups were as follows: the control group (C), which was given a normal diet containing 10% of total calories from fat; the HFD group (HFDs contain 36% of total calories from fat) [33]; the S40 group (40 mg/kg BW of simvastatin plus HFD); the PF250 group (250 mg/kg BW of PF extract plus HFD); the PF500 group (500 mg/kg BW of PF extract plus HFD). Both PF extract and simvastatin were dissolved in 1 mL of distilled water and administered to the rats via oral gavage once a day for 8 weeks. Food consumption was recorded daily and the BW of the rats were measured and recorded weekly. After 8 weeks, the rats’ gastrocnemius (GAS) muscles were collected under anesthesia after a 12‐h fast. The rats were then euthanized. The muscle samples were homogenized in 0.1 M PBS using a sonicator and then centrifuged at 1000 g for 20 min at 4°C to separate the supernatants which were stored at −80°C for further analysis.

### 2.8. Adiposity Index

The total body fat was calculated using the weights of apart fat pads: epididymal fat + retroperitoneal fat + visceral fat. The adiposity index was calculated as (total body fat/final BW) × 100 [34].

### 2.9. Oxidative Status

#### 2.9.1. Malondialdehyde (MDA)

MDA levels were determined as a measure of lipid peroxidation in the GAS muscle. In brief, the ratio 1:1:1 (*V*:*V*:*V*) of 0.37% of thiobarbituric acid (TBA), 15% *w*/*w* trichloroacetic acid, and 0.25 N hydrochloric acid (HCl) were added into the tube. After that, the solution was mixed with the muscle homogenate and incubated for 15 min. The samples were centrifuged at 25°C at 3500 rpm, and their absorbance was measured at 532 nm.

#### 2.9.2. SOD

A 4 µL of muscle homogenate was loaded into the cuvette. After that, the mixture, 470 µL of Tris buffer, 360 µL of distilled water, and 4.5 mmol/L of pyrogallol, was loaded in 10 mmol/L of HCl. After mixing, the wavelength was measured at 325 nm using a spectrophotometer.

#### 2.9.3. CAT

The cuvette was filled with 10 µL of sample, 50 mM PBS, and 30% hydrogen peroxide (H_2_O_2_). After that, the solution was mixed and measured at 240 nm.

#### 2.9.4. GPx

The samples were added with 0.2 M phosphate buffer (pH 8.0), 10 mM sodium azide (NaN_3_), 4 mM reduced glutathione (GSH), 2.5 mM H_2_O_2_, 5% trichloroacetic acid, 1.2 mM 5,5^′^‐dithiobis‐(2‐nitrobenzoic acid) (DTNB), and distilled water. The sample was mixed with H_2_O_2_ and GSH, phosphate buffer, and NaN_3_. After that, a mixed solution was incubated at 37°C. Following that, add 5% trichloroacetic acid and spin down for 1 min. The supernatant was collected, and 50 µL of DTNB was added. The absorbance at 412 nm was measured and compared to a blank solution.

### 2.10. Lipid Profiles

#### 2.10.1. FFAs Levels

FFAs levels were assessed using methods adapted from a prior study [35]. In summary, 50 μL of serum was added to 1 mL of copper acetate. Then, the solution was mixed and centrifuged at 3000 × *g* for 5 min. The pellet was dissolved with 1% pyridine in chloroform. Before measuring the absorbance at 715 nm, the solution was centrifuged at 10,000 rpm at 4°C, and the solution phase was determined.

#### 2.10.2. TGs and TC Levels

A 1:2 (*v*:*v*) methanol:chloroform solution was used to extract 100 mg of tissue, which was homogenized in the tube using a sonicator and centrifuged at 1000 × *g* for 5 min. Then, the supernatant was removed, and the remaining portion was nitrogen‐dried and dissolved with hexane. The levels of TG and TC in the GAS muscle were measured using commercial assay kits (HUMAN Gesellschaft fur Biochemica und Diagnostics mbH, Wiesbaden, Germany).

### 2.11. Western Blot Analysis

The expressions of Nrf2, IRS‐1, NF‐κB, phosphorylated AKT (p‐AKT), and total AKT (t‐AKT) in proteins were detected using a Western blot analysis. The 60 µg protein samples were separated using sodium dodecyl sulfate‐polyacrylamide gels before being transferred to polyvinylidene fluoride (PVDF) membranes. The membrane was washed with 1x of TBST after being incubated with 5% skim milk. The membranes were coated with primary antibodies Nrf2, IRS‐1, t‐AKT, p‐AKT, NF‐κB, and β‐actin at different dilutions (Supporting Information 1: Table S1) and incubated overnight at 4°C. Subsequently, they were incubated with horseradish peroxidase‐conjugated anti‐mouse for an hour, and the protein expression was detected using a chemiluminescence machine (ImageQuant LAS 500, GE Healthcare Bio‐Sciences AB, Uppsala, Sweden). The bands were measured and the relative expressions of the proteins were compared using the Image Lab program. The expression of Nrf2, IRS‐1, and NF‐κB was normalized by β‐actin, whereas p‐AKT was normalized by t‐AKT.

### 2.12. Glucose Uptake

The method was adapted from previous research [36]. The small sections of GAS muscle were transferred into Krebs‐Ringer bicarbonate (KRB) buffer solution of O_2_ and CO_2_ at 37°C. After that, the muscles were transferred into a 1.5 mL Eppendorf tube that contained a KRB solution with glucose, insulin, and without insulin. The Glucose (GO) assay kit (Sigma–Aldrich, St. Louis, MO, USA) was used to measure glucose concentrations.

### 2.13. Glycogen

The muscle glycogen levels were measured using a technique adapted from the previous study [37]. In brief, 150 mg of tissue was boiled in potassium hydroxide (KOH). Then, 95% ethanol was added and incubated on ice. Samples were centrifuged and collected supernatant then added distilled water, 95% sulfuric acid, and incubated for 30 min. GO assay kit (Sigma–Aldrich, St. Louis, MO, USA) was used to measure glycogen levels at a wavelength of 540 nm.

### 2.14. Muscle Injury

#### 2.14.1. Creatine Kinase (CK) Levels

A reconstituted reagent (assay buffer, substrate solution, and enzyme mix) was added to separate wells, followed by 10 µL of samples. After that, the solution was mixed and the plate was incubated at room temperature. The initial absorbance of CK levels was measured at 340 nm. Continue to incubate at room temperature for 20 min before measuring the final absorbance (Sigma–Aldrich, St. Louis, MO, USA).

#### 2.14.2. Lactate Dehydrogenase (LDH) Levels

The LDH levels were measured using a LDH activity assay kit from Sigma–Aldrich. Samples were mixed with the master reaction mix and absorbance was measured at 450 nm. Subsequently, the plate was incubated at 37°C and absorbance was recorded every 5 min until the sample’s value exceeded the highest standard’s value. Finally, the activity was calculated based on the final absorbance reading.

### 2.15. Muscle Inflammation

IL‐6 and TNF‐α were determined using related ELISA kits following the guidelines from the manufacturer (Sigma–Aldrich, St. Louis, MO, USA). In brief, 100 µL of the sample was added to the well plate, followed by a 20x wash buffer. After that, incubated at room temperature with the detection antibody. The last step was incubated in the dark for 30 min with HRP–streptavidin. The stop reaction solution was added and the samples were measured for the OD at 450 nm.

### 2.16. Real‐Time Polymerase Chain Reaction

TRI Reagent (Molecular Research Center, Inc.) was used to isolate total RNA from the GAS muscle. DNase I reaction and reverse transcription were used to convert total RNA to cDNA. The primer sets for *Trim63*, *Fbxo32*, *Mtor*, *Cd36*, *Pi3k*, *Bax*, *Bcl2*, *Slc2a4*, *Hmgcr*, and glyceraldehyde‐3‐phosphate dehydrogenase (*Gapdh*) were used in real‐time PCR reactions, which were carried out using PCR thermocycling machines. The mRNA expression levels of the target genes were normalized to *Gapdh* expression levels. The primer sequences are provided in Supporting Information 1: Table S2.

### 2.17. Histopathology

The GAS muscle and white adipose tissues were fixed with 10% neutral buffer formalin before being processed according to a standard protocol and embedded in a paraffin block. The block was cut into 5 µm thick sections and put on the slides. Slides were then stained using hematoxylin and eosin (H&E). Image J was used to measure the images that were obtained using a light microscope. The quantity, the smallest Feret’s diameter, and the cross‐sectional area (CSA) of muscle fibers per unit area were employed to depict the morphology of muscle fibers. The size and area of white adipose tissues were measured using the same Image J procedures as muscle. All measurements were conducted in five randomly chosen fields of each section from three sections per rat (five rats/group).

### 2.18. ATP Content

ATP content was assessed using an ATP colorimetric assay kit according to the manufacturer’s instructions (Elabscience, Houston, USA). In brief, 20 mg of GAS was extracted with an extracting solution. After that, the samples were homogenized and centrifuged at 10,000 × g, and the supernatants were collected. The samples or 1 mmol/L ATP were added to the tube using 30 and 330 µL of detection or control workings. The mixture was incubated at 37°C before adding protein precipitator. The samples were centrifuged at 10,000 × g before adding supernatant and chromogenic agent, and mixed before adding 500 µL of stop solution, and waiting 5 min. The samples were measured for OD at 636 nm.

### 2.19. Statistical Analysis

One‐way analysis of Variance (ANOVA) followed by Tukey’s multiple comparison test was used to analyze all the data using GraphPad Prism 8 (GraphPad Software Inc., San Diego, CA, USA), and the results were presented as the mean and standard error of the mean (SEM). A statistically significant difference was evaluated as *p*  < 0.05.

## 3. Results

### 3.1. The Evaluation of Gallic Acid and Caffeic Acid in the PF Extract

**Figure 1:**
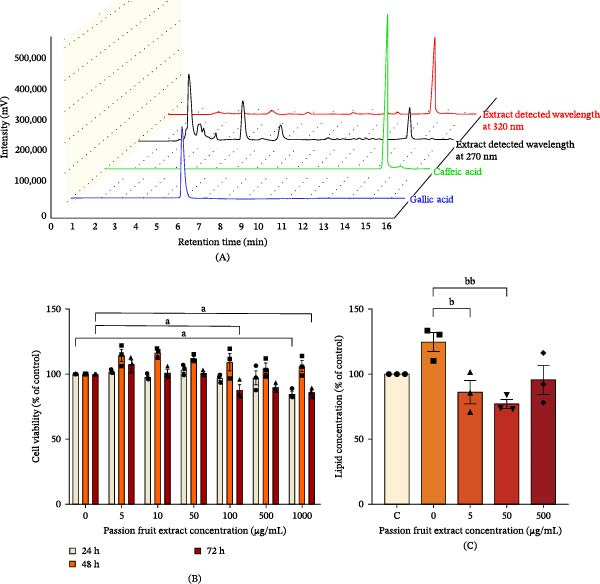
The active compounds and impact of PF extract on cell viability and lipogenesis in 3T3‐L1 cells. (A) The phenolic compounds in the PF extract at 100 mg/mL, (B) the viability, and (C) fat deposition in 3T3‐L1 cells. Results are shown as mean ± SEM (*n* = 3). One‐way ANOVA was used to evaluate group differences, followed by Tukey’s multiple comparison test (*p* < 0.05). The letters represented “a” compared to the untreated group, “b” compared to the induction group, “c” compared to the 5 μg/mL group, “d” compared to the 50 μg/mL group, and “e” compared to the 500 μg/mL group. *Note:* Single, double, and triple letters represent *p* < 0.05, *p* < 0.01, and *p* < 0.001, respectively. ^a^ *p* < 0.05 compared with the untreated group at 24 or 72 h. ^b^ *p* < 0.05 and ^bb^ *p* < 0.01 compared with the induction group.

Both biomarkers were identified using the HPLC technique, which demonstrated clear chromatographic separation along with good peak resolution. The biomarkers eluted with retention times of 5.3 min for gallic acid and 13.7 min for caffeic acid. Additionally, the concentration ranges of 1.0–50.0 µg/mL exhibited excellent linearity of calibration curves and correlation coefficients (*R* ^2^), which indicates high reliability of the quantitative analysis (Supporting Information 2: Figure S1). The chromatogram of the PF extract exhibited identical major peaks for gallic acid and caffeic acid at the same retention periods as the reference standards, confirming the presence of both compounds in the extract. According to the findings, the PF extract contained 305.2 ± 4.86 µg/g of gallic acid and 320.5 ± 4.90 µg/g of caffeic acid (Figure 1A).

### 3.2. *In Vitro* Ability to Inhibit Cholesterol Micellar Solubility of PF Extract

The results showed that PF extract was an inhibitor of cholesterol micellar solubility, with an IC_50_ value of 3431 µg/mL and an inhibition range of 5%–62% in dose‐dependent extracts (250, 500, 1000, and 5000 µg/mL). On the other hand, gallic acid demonstrated inhibitory properties with an IC_50_ value of 580.8 µg/mL and a range of inhibition of 17%–87% in dose‐dependent gallic acids (62.5, 125, 250, 500,1000, 5000, and 10,000 µg/mL). This study found that PF extract may have a role in reduced cholesterol absorption through inhibited cholesterol micellar solubility.

### 3.3. Cytotoxicity of PF Extract in 3T3‐L1 Cells

The MTT assay was used to assess the viability of 3T3‐L1 preadipocytes after exposure to PF extract (5–1000 μg/mL) for 24, 48, and 72 h. The PF extract at dosages of 5, 10, 50, and 500 μg/mL for 24 and 48 h exhibited no change in the viability of 3T3‐L1, while a slight decline at 500 and 1000 µg/mL for 72 h was observed compared to the control group (Figure 1B). Thus, the extract with the highest concentration, 5, 50, and 500 μg/mL, was chosen for further study.

### 3.4. The PF Extract Suppressed Adipogenesis in 3T3‐L1 Cells

After treatment with a hormone combination consisting of dexamethasone, methyl isobutyl xanthine, and insulin. The induced 3T3‐L1 preadipocyte cells showed a tendency to enhance fat deposition, although the difference was not significant when compared to the control. However, PF extracts at 5 and 50 μg/mL exhibited a reduction in fat accumulation when compared to the induction group (Figure 1C).

### 3.5. The PF Extract Prevented Obesity in HFD–Fed Rats

All groups obtained equal initial BW, however by week 8, the rats that were given an HFD experienced a significant increase in BW by about 30% compared to the C group. Rats that were administered PF extract and simvastatin exhibited a decrease in BW and BW gain compared to the HFD group (Table 1). Similar to BW, the adiposity index increased in the HFD group while decreased in the PF250, PF500, and S40 groups. The results indicated that PF extract has a protective effect against BW gain in rats that were fed an HFD, as evidenced by a reduction in the adiposity index (Table 1). The findings demonstrated that PF extract and simvastatin can help prevent obesity caused by HFD.

**Table 1:** The effects of PF extract on body weight, the percentage of body weight gain, fat weight, and adiposity index in rats fed the normal diet and high‐fat diet for 8 weeks.

Parameters	Animal groups				
	C	HFD	PF250	PF500	S40
Body weight week 0 (g)	295.70 ± 5.13	305.60 ± 6.02	296.30 ± 3.73	302.70 ± 5.11	308.00 ± 5.09
Body weight week 8 (g)	500.10 ± 11.45	651.40 ± 10.55^aaa^	573.60 ± 16.59^aa, bb^	555.80 ± 9.71^a,bbb^	544.30 ± 10.62^bbb^
Percentage gain in body weight at week 8 (in comparison to week 0)	69.51 ± 5.21	113.30 ± 2.52^aaa^	93.46 ± 4.14^aaa,bb^	83.81 ± 3.66^bbb^	76.80 ± 2.79^bbb,c^
Epididymal fat (g)	4.02 ± 0.22	6.17 ± 0.29^aaa^	4.65 ± 0.42^bb^	3.93 ± 0.20^bbb^	4.03 ± 0.20^bbb^
Retroperitoneal fat (g)	4.97±0.12	8.81 ± 0.39^aaa^	6.30 ± 0.42^bbb^	5.31 ± 0.25^bbb^	4.82 ± 0.45^bbb,c^
Visceral fat (g)	8.19 ± 0.51	14.47 ± 0.79^aaa^	8.92 ± 0.57^bbb^	8.39 ± 0.78^bbb^	9.12 ± 0.41^bbb^
Adiposity index	4.28 ± 0.19	5.84 ± 0.28^aaa^	4.37 ± 0.23^bbb^	3.64 ± 0.13^bbb^	4.07 ± 0.18^bbb^

*Note:* Data are presented as mean ± SEM (*n* = 7). One‐way ANOVA was used to evaluate group differences, followed by Tukey’s multiple comparison tests (*p* < 0.05). The letters represented “a” compared to the control group, “b” compared to the HFD group, “c” compared to the PF250 group, and “d” compared to the PF500 group.

^a^ *p* < 0.05, ^aa^ *p* < 0.01, ^aaa^ *p* < 0.001 compared with C group.

^bb^ *p* < 0.01 and ^bbb^ *p* < 0.001 compared with HFD group.

^c^ *p* < 0.05 compared with PF250 group.

### 3.6. The PF Extract Decreased Lipid Accumulation in the Skeletal Muscle and Adipose Tissues

**Figure 2:**
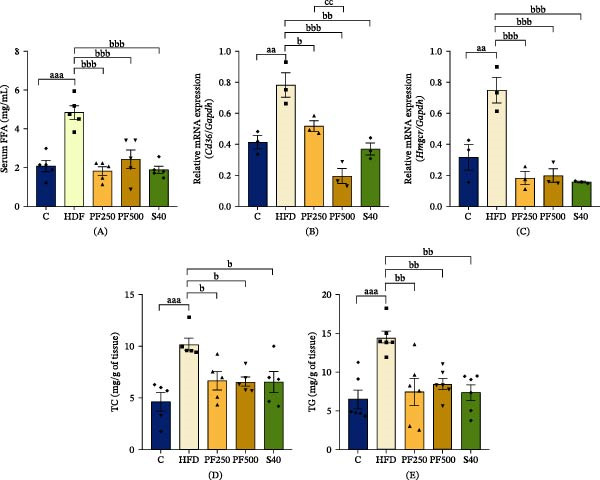
The effect of PF extract on lipid accumulation in the skeletal muscle of high‐fat diet‐induced obese rats. (A) The FFAs levels in the serum (*n* = 5). (B) The expression of *Cd36* in the gastrocnemius muscle was determined by RT‐PCR (*n* = 3). (C) The expression of *Hmgcr* in the gastrocnemius muscle was determined by RT‐PCR (*n* = 3). (D) The levels of TC (*n* = 5) and (E) TG (*n* = 6) in the gastrocnemius muscle. Results are shown as mean ± SEM. One‐way ANOVA was used to evaluate group differences, followed by Tukey’s multiple comparison test (*p* < 0.05). The letters represented “a” compared to the control group, “b” compared to the HFD group, “c” compared to the PF250 group, and “d” compared to the PF500 group. ^aa^ *p* < 0.01, and ^aaa^ *p* < 0.001 compared with the C group. ^b^ *p* < 0.05, ^bb^ *p* < 0.01 and ^bbb^ *p* < 0.001 compared with the HFD group. ^cc^ *p* < 0.01 compared with the PF250 group.

**Figure 3:**
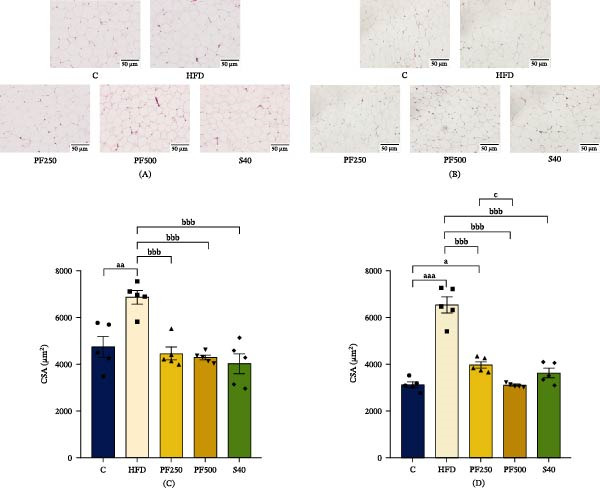
The impact of PF extract on lipid accumulation in adipose tissues of obese rats fed a high‐fat diet. (A) Representative images of the epididymal and (B) perirenal adipose tissues along with histological sections after staining with hematoxylin and eosin (H&E), scale bar = 50 µm. The size of (C) epididymal and (D) perirenal tissues. Results are shown as mean ± SEM (*n* = 5). One‐way ANOVA was used to evaluate group differences, followed by Tukey’s multiple comparison test (*p* < 0.05). The letters represented “a” compared to the control group, “b” compared to the HFD group, “c” compared to the PF250 group, and “d” compared to the PF500 group. ^a^ *p* < 0.05 and ^aa^ *p* < 0.01, and ^aaa^ *p* < 0.001 compared with the C group. ^bbb^ *p* < 0.001 compared with the HFD group. ^c^ *p* < 0.05 compared with the PF250 group.

To determine the effect of PF extract on lipid accumulation, FFAs levels, adipose tissue weight, the expression of *Cd36*, and fat accumulation levels in skeletal muscle were assessed. Table 1 shows that the adipose tissue weight and adiposity index increased in the HFD group while reducing in the treatment groups. Furthermore, FFAs levels in serum increased in the HFD group while decreasing in the treatment groups, coinciding with fat absorption (Figure 2A). As shown in Figure 2B, *Cd36*, which is associated with fatty acid transport into cells, and *Hmgcr*, an enzyme involved in cholesterol biosynthesis are upregulated in the HFD group (Figure 2C). This finding takes part in an increase in TC and TG levels in the HFD group (Figure 2D,E). However, the administration of PF extract and simvastatin was shown to significantly inhibit fatty acid absorption and lipid synthesis in skeletal muscle. Moreover, HFD–fed rats showed increased adipocyte sizes compared with the C group whereas PF extract and simvastatin decreased the size of adipocytes (Figure 3A–D). These results demonstrated that administering PF extract and simvastatin decreased lipid accumulation in rats fed a high‐fat diet.

### 3.7. The PF Extract Improved Oxidative Status by Increasing Nrf2 Expression in the GAS Muscles

**Figure 4:**
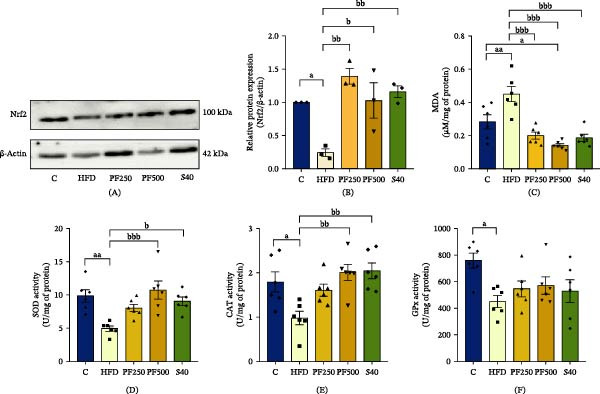
The effect of PF extract on muscle oxidative status in high‐fat diet‐induced obese rats. (A) The expression of Nrf2 in the gastrocnemius muscle was determined by western blot. (B) Nrf2 levels were quantified (*n* = 3). (C) MDA levels. (D) SOD activity. (E) CAT activity, and (F) GPx activity. Results are shown as mean±SEM (C–F, *n* = 6). One‐way ANOVA was used to evaluate group differences, followed by Tukey’s multiple comparison test (*p* < 0.05). The letters represented “a” compared to the control group, “b” compared to the HFD group, “c” compared to the PF250 group, and “d” compared to the PF500 group. ^a^ *p* < 0.05 and ^aa^ *p* < 0.01 compared with the C group. ^b^ *p* < 0.05, ^bb^ *p* < 0.01, and ^bbb^ *p* < 0.001 compared with the HFD group.

The expression of Nrf2 in skeletal muscle tissues was evaluated by Western blot analysis to investigate the effects of PF extract on antioxidant enzymes and lipid peroxidation, which is an important consideration for oxidative status. The HFD group exhibited decreased Nrf2 expression levels in muscle compared to the C group, while the PF250, PF500, and S40 groups demonstrated a notable rise (Figure 4A,B). The results indicated that the PF extract and simvastatin significantly increased Nrf2 expression in the skeletal muscle. Hence, PF extract possibly increased antioxidant enzymes while decreasing lipid peroxidation by activating Nrf2 expression. The levels of lipid peroxidation and antioxidant enzymes including MDA and CAT, SOD, and GPx, respectively, in skeletal muscle were measured using commercial kits in this study. MDA levels increased in the HFD group whereas decreased in the treatment groups (Figure 4C). In the same way as antioxidant enzymes (CAT, SOD, and GPx) levels significantly decreased in the HFD group when compared with the C group (Figure 4D–F). On the other hand, S40 and PF500 groups showed an increase in SOD and CAT activity (Figure 4D,E). From the results, the 500 mg/kg of PF extract decreased the MDA level while increasing CAT and SOD activity in the GAS muscle. Together, these results showed that PF extracts and simvastatin improved antioxidant status and decreased oxidative stress by activating Nrf2 expression in HFD‐induced muscle atrophy.

### 3.8. The PF Extract Promoted Skeletal Muscle Glucose Metabolism Through the IRS‐1/PI3K/AKT Signaling Pathway

**Figure 5:**
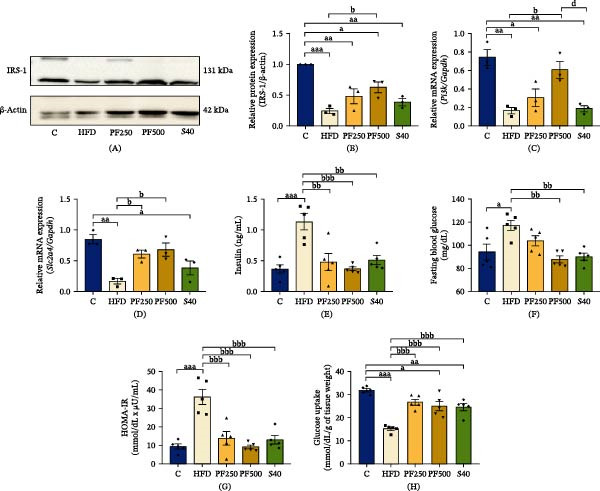
The effect of PF extract on glucose metabolism in high‐fat diet‐induced obese rats. (A) The expression of IRS‐1 in the gastrocnemius muscle was determined by western blot. (B) IRS‐1 levels were quantified (*n* = 3). (C) The expression of *Pi3k* in the gastrocnemius muscle was determined by RT‐PCR (*n* = 3). (D) The expression of *Slc2a4* in the gastrocnemius muscle was determined by RT‐PCR (*n* = 3). (E) Insulin. (F) Fasting blood glucose. (G) HOMA‐IR and (H) glucose uptake. Results are shown as mean ± SEM (E–H, *n* = 5). One‐way ANOVA was used to evaluate group differences, followed by Tukey’s multiple comparison test (*p* < 0.05). The letters represented “a” compared to the control group, “b” compared to the HFD group, “c” compared to the PF250 group, and “d” compared to the PF500 group. ^a^ *p* < 0.05, ^aa^ *p* < 0.01, and ^aaa^ *p* < 0.001 compared with the C group. ^b^ *p* < 0.05, ^bb^ *p* < 0.01, and ^bbb^ *p* < 0.001 compared with the HFD group. ^d^ *p* < 0.05 compared with the PF500 group.

**Figure 6:**
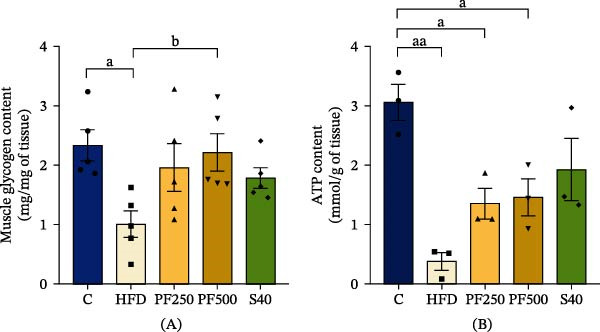
The effect of PF extract on glycogen and ATP contents in high‐fat diet‐induced obese rats. (A) Muscle glycogen content (*n* = 5) and (B) muscle ATP content (*n* = 3). Results are shown as mean ± SEM. One‐way ANOVA was used to evaluate group differences, followed by Tukey’s multiple comparison test (*p* < 0.05). The letters represented “a” compared to the control group, “b” compared to the HFD group, “c” compared to the PF250 group, and “d” compared to the PF500 group. ^a^ *p* < 0.05 and ^aa^ *p* < 0.01 compared with the C group. ^b^ *p* < 0.05 compared with the HFD group.

To examine the changes in insulin sensitivity caused by an HFD. IRS‐1, *Pi3k*, p‐AKT, and *Slc2a4* expressions were examined, as well as the function that is important to glucose uptake regulation. HFD had a propensity to reduce IRS‐1, *Pi3k*, p‐AKT, and *Slc2a4* expressions when compared with the C group (Figures 5A–D). This is in accordance with insulin levels, fasted blood glucose (FBG), and homeostasis model assessment of IR (HOMA‐IR), which showed an increase in the HFD group but decreased glucose absorption (Figure 5E–H). However, high dose of PF extract improves glucose absorption in skeletal muscle through increased IRS‐1, *Pi3k*, p‐AKT, and *Slc2a4* expressions, and the insulin levels, FBG, and HOMA‐IR were reduced in the PF extract and simvastatin groups. Furthermore, glycogen and ATP in skeletal muscle are reduced in the same direction as glucose in the HFD group, but it was only found to increase after receiving 500 mg/kg of PF extract (Figure 6A,B). Our findings demonstrated that PF extract prevented IR and improved glucose metabolism by promoting the IRS‐1/PI3K/p‐AKT signaling pathway and *Slc2a4* expression.

### 3.9. The PF Extract Improved Skeletal Muscle Energy Metabolism and Injury in the HFD‐Fed Rats

CK and LDH are enzymes in skeletal muscle energy metabolism. In this study, both enzymes have been found to be higher in the HFD group than in the control group due to metabolic stress. However, the PF extract and simvastatin returned both enzyme levels to normal (Table 2). Thus, our findings suggested that PF extracts help to recover skeletal muscle energy metabolism to the normal level. Additionally, CK and LDH in serum are common tissue damage indicators. Table 2 shows the CK and LDH activities in the HFD group were significantly increased compared to the C group, whereas the activity in PF250, PF500, and S40 groups significantly decreased compared with the HFD group. On the other hand, LDH activity tends to decrease in the PF500 group. Thus, these results indicated that PF extracts ameliorated muscle injury in HFD‐induced obese rats.

**Table 2:** The effects of PF extract on skeletal muscle energy metabolism and muscle injury in rats fed the normal diet and high‐fat diet for 8 weeks.

Parameters	Animal groups				
	C	HFD	PF250	PF500	S40
Serum CK (U/L)	457.00 ± 157.5	914.00 ± 37.65^a^	357.30 ± 76.13^bb^	187.00 ± 43.09^bbb^	325.00 ± 33.08^bb^
Serum LDH (U/L)	253.30 ± 39.94	1299.00 ± 131.60^aaa^	256.7 ± 50.67^bbb^	354.30 ± 124.90^bbb^	345.00 ± 121.2^bbb^
Muscle CK (U/mL/mg protein)	31.12 ± 1.98	42.37 ± 2.44^a^	26.67 ± 1.79^bb^	31.24 ± 2.80^b^	27.97 ± 3.43^bb^
Muscle LDH (U/mL/mg protein)	57.04 ± 4.46	93.87 ± 11.02^aa^	54.17 ± 3.41^bb^	74.58 ± 5.19	50.07 ± 6.04^bbb^

*Note:* Data are presented as mean ± SEM. The CK and LDH levels in muscle (*n* = 6) and serum (*n* = 3). One‐way ANOVA was used to evaluate group differences, followed by Tukey’s multiple comparison tests (*p* < 0.05). The letters represented “a” compared to the control group, “b” compared to the HFD group, “c” compared to the PF250 group, and “d” compared to the PF500 group.

^a^ *p* < 0.05, ^aa^ *p* < 0.01, ^aaa^ *p* < 0.001 compared with C group.

^b^ *p* < 0.05, ^bb^ *p* < 0.01, ^bbb^ *p* < 0.001 compared with HFD group.

### 3.10. The PF Extract Reduced Inflammatory Responses in the Muscle of HFD‐Induced Obese Rats via Inhibiting the Expression of NF‐κB

**Figure 7:**
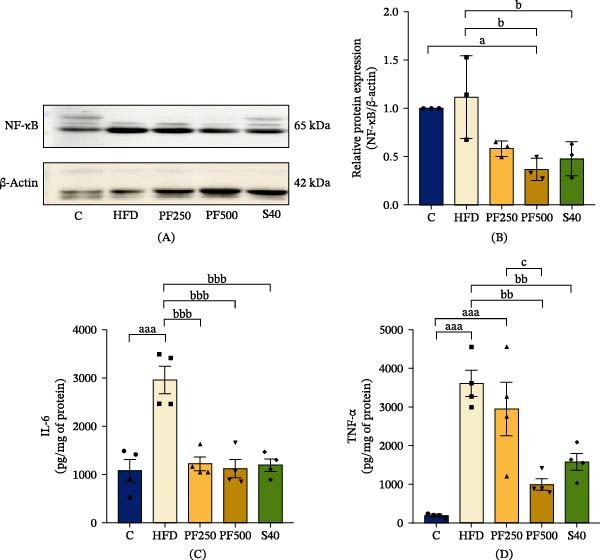
The effect of passion fruit extract on muscle inflammation in high‐fat diet‐induced obese rats. (A) The expression of NF‐кB in the gastrocnemius muscle was determined by western blot. (B) NF‐кB levels were quantified (*n* = 3). (C) IL‐6 level and (D) TNF‐α level. Results are shown as mean ± SEM (C, D, *n* = 4). One‐way ANOVA was used to evaluate group differences, followed by Tukey’s multiple comparison test (*p* < 0.05). The letters represented “a” compared to the control group, “b” compared to the HFD group, “c” compared to the PF250 group, and “d” compared to the PF500 group. ^a^ *p* < 0.05, and ^aaa^ *p* < 0.001 compared with the C group. ^b^ *p* < 0.05, ^bb^ *p* < 0.01, and ^bbb^ *p* < 0.001 compared with the HFD group. ^c^ *p* < 0.05 compared with the PF250 group.

Ectopic lipid accumulation in peripheral organs is a normal contributor to chronic inflammation. NF‐κB tended to increase in the HFD group (Figure 7A,B), leading to an increase in proinflammatory cytokines such as IL‐6 and TNF‐α (Figure 7C,D). In contrast, the PF500 and S40 groups showed a decrease in both cytokines. Thus, both PF extract and simvastatin can exhibit an anti‐inflammatory property via inhibiting NF‐κB expression.

### 3.11. The PF Extract Protected Against HFD‐Induced Muscle Atrophy via Decreasing Protein Degradation

**Figure 8:**
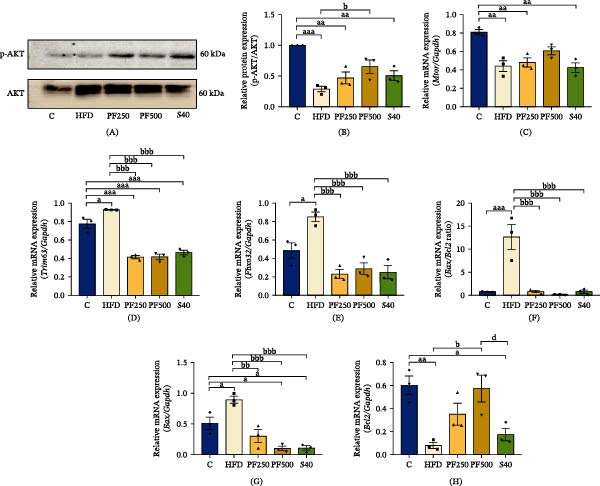
The effect of PF extract on gene and protein expression‐related muscle atrophy in high‐fat diet–induced obese rats. (A) The expression of p‐AKT in the gastrocnemius muscle was determined by western blot. (B) p‐AKT levels were normalized with t‐Akt and the p‐AKT/t‐AKT ratio was quantified. (C) The expression of *Mtor* in the gastrocnemius muscle was determined by RT‐PCR. (D) The expression of *Trim63* in the gastrocnemius muscle was determined by RT‐PCR. (E) The expression of *Fbxo32* in the gastrocnemius muscle was determined by RT‐PCR. (F) The ratio of *Bax/Bcl2*. (G) The expression of *Bax* in the gastrocnemius muscle was determined by RT‐PCR. (H) The expression of *Bcl2* in the gastrocnemius muscle was determined by RT‐PCR. Results are shown as mean ± SEM (*n* = 3). One‐way ANOVA was used to evaluate group differences, followed by Tukey’s multiple comparison test (*p* < 0.05). The letters represented “a” compared to the control group, “b” compared to the HFD group, “c” compared to the PF250 group, and “d” compared to the PF500 group. ^a^ *p* < 0.05, ^aa^ *p* < 0.01, and ^aaa^ *p* < 0.001 compared with the C group. ^b^ *p* < 0.05, ^bb^ *p* < 0.01, and ^bbb^ *p* < 0.001 compared with the HFD group. ^d^ *p* < 0.05 compared with the PF500 group.

**Figure 9:**
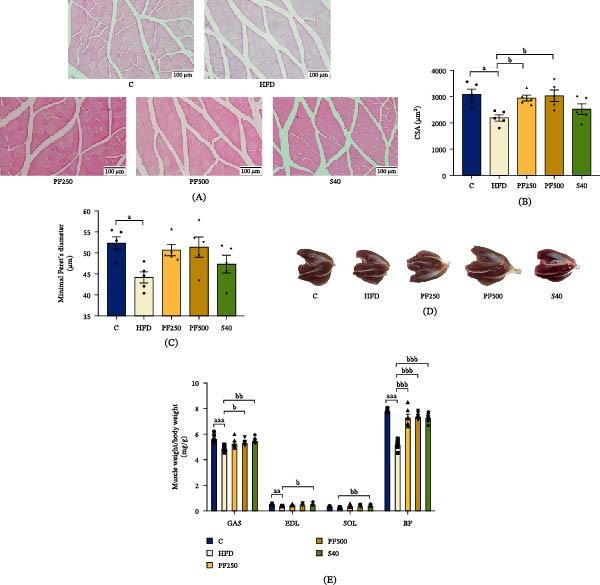
The effect of PF extract on muscle atrophy in high‐fat diet‐induced obese rats. (A) Representative images of the gastrocnemius muscle along with histological sections after staining with hematoxylin and eosin (H&E), Scale bar = 100 µm. (B) The CSA of the gastrocnemius muscle. (C) Minimal Feret’s diameter of the gastrocnemius muscle. (D) Gross morphological of the gastrocnemius muscle. (E) Muscle weight/body weight. Results are shown as mean ± SEM (B, C, *n* = 5 and E, *n* = 7). One‐way ANOVA was used to evaluate group differences, followed by Tukey’s multiple comparison test (*p* < 0.05). The letters represented “a” compared to the control group, “b” compared to the HFD group, “c” compared to the PF250 group, and “d” compared to the PF500 group. ^a^ *p* < 0.05, ^aa^ *p* < 0.01, and ^aaa^ *p* < 0.001 compared with the C group. ^b^ *p* < 0.05, ^bb^ *p* < 0.01, and ^bbb^ *p* < 0.001 compared with the HFD group. BF; bicep femoris; EDL, extensor digitorum longus; GAS, gastrocnemius; SOL, soleus.

Imbalances in protein synthesis and breakdown are associated with muscle loss. To determine that PF extract improved muscle atrophy, we assessed the expression of important mediators of muscular protein synthesis and breakdown. In the HFD group, the expressions of *Trim63* and *Fbxo32* were significantly increased while *Pi3k*, p‐AKT, and *Mtor* expressions were decreased when compared with the C group (Figures 5C and 8A–E). Furthermore, the diameter and CSA decreased in the HFD group (Figure 9B,C). However, PF extract has demonstrated an improved imbalance of protein breakdown and synthesis by suppressing the expression of *Trim63* and *Fbxo32*, while enhancing the PI3K/AKT/MTOR signaling pathway. As a result, the fiber diameter and CSA increased. These results contributed to hind‐limb muscle weight, particularly GAS and bicep femoris (BF) muscles, which were significantly decreased in the HFD group but recovered in the PF250, PF500, and S40 groups (Figure 9E). Overall, these findings suggested that PF extract can prevent HFD‐induced muscle atrophy by decreasing the degradation of proteins.

### 3.12. The PF Extract Modulated the Apoptosis‐Related Muscle Atrophy in HFD‐Induced Obese Rats

*Bax* is a proapoptotic gene that regulates the release of cytochrome C, triggering apoptosis. *Bcl2* is an antiapoptotic gene that prevents the release of cytochrome C. To assess the effects of PF extract on muscle cell apoptosis, *Bax* and *Bcl2* expression levels were measured. The HFD group showed an increase in *Bax* expression while a decrease in *Bcl2* expression when compared with the C group (Figure 8G,H). When apoptosis was stimulated in the HFD group, the *Bax*/*Bcl2* ratio increased (Figure 8F). Meanwhile, PF extract and simvastatin suppressed *Bax* expression, but only PF extract improved *Bcl2* expression. However, both PF extract and simvastatin showed a decrease in the *Bax*/*Bcl2* ratio. Thus, the findings may indicate that PF extract and simvastatin can inhibit mitochondrial apoptosis‐induced muscle atrophy.

## 4. Discussion

High energy consumption increases lipid accumulation in adipocytes, which is positively associated with the prevalence of obesity [38]. Weight gain, body fat mass, and other parameters such as adiponectin, mesenteric adipocyte size, and others increased in HFD rats [39]. Our findings showed that adipocyte cells tend to accumulate lipids, whereas PF extract inhibits fat formation in adipocyte cells. This demonstrated that PF extract might have the ability to inhibit fat accumulation. Additionally, previous reports show that rats given an HFD gained approximately 10% more weight than those fed a normal diet, which is a hallmark of obesity [40], in accordance with the model used in this study. HFD–induced obesity increased not only BW, but also adipocyte size, fat weight, and adiposity index. Nonetheless, the final BW, adipocyte size, and adiposity index were diminished in the treatment groups compared to the HFD group. The adiposity index decreased in the treatment groups, possibly due to loss of fat pad weights rather than muscle preservation [41]. Consequently, the results indicate that the use of PF extract and simvastatin may aid in the prevention of weight gain and the reduction of fat accumulation. These findings align with the previous outcomes, which reported that *P. edulis* peel flour (PEPF) decreased the absorption of glucose and lipids, leading to a reduction in energy storage, and account for a decrease in weight gain and fat mass [42]. Nevertheless, fat may still accumulate in non‐adipose tissue, known as ectopic fat accumulation, which is caused by heightened absorption of FFAs, increased synthesis, or reduced oxidation of FFAs. A prior study found that higher fatty acid transporters in obese rats promote an increase in triacylglycerol concentration in the muscle [43]. Similarly, our study revealed an elevation in *Cd36* expression in the HFD group. Additionally, higher levels of TG and TC were noted in the GAS muscle of obese rats compared to normal rats in our study. Conversely, the PF extract and simvastatin groups showed reduced fatty acid transporter expression, resulting in decreased fat uptake into muscle cells. These findings were similar to those shown in previous research which had reported that piceatannol, an active compound found in passion fruit, reduces fat accumulation by decreasing FFAs absorption and lipogenesis by downregulating CD36 expression, sterol regulatory element binding protein 1 (SREBP1), and acetyl‐coa carboxylase (ACC) while increasing FFAs oxidation [44]. Also, we found that PF extract suppresses *Hmgcr* expression, leading to decreased lipid accumulation, in the same way as simvastatin works. These data indicated that both dosages of PF extract decreased fat accumulation via reducing fatty acid transport and synthesis. In addition, the micellar solubilization of cholesterol is crucial for absorption in humans [45]. Inhibition of fat absorption from the small intestine is an attractive avenue for dyslipidemia treatment [46]. Our findings indicated that the PF extract has the ability to diminish micellar solubility, which might minimize cholesterol absorption and accumulation in other organs. Similarly, a previous study found that limiting cholesterol micellar solubility reduces cholesterol absorption into intestinal cells [47].

Excessive fat consumption causes lipid accumulation and develops into lipotoxicity, which affects normal muscle function [48]. The oxidation of lipids produces ROS, which triggers lipid peroxidation, culminating in tissue injury, a process that can be determined through the measurement of MDA levels. MDA, as a by‐product of lipid peroxidation, serves as a valuable biomarker for assessing oxidative stress [49]. Nrf2 regulates the antioxidant defense system by upregulating antioxidant enzymes such as SOD, CAT, and GPx, which protect against ROS‐induced cellular damage [50]. We found that HFD suppressed Nrf2 expression, leading to reduced SOD, CAT, and GPx levels, but increased MDA levels in GAS muscle, whereas PF extract and simvastatin resolved these defects. These findings are consistent with previous studies that show HFD causes oxidative stress by suppressing Nrf2 expression in skeletal muscle [51]. In line with our discoveries, we have observed that PF extract possesses antioxidant properties that contribute to the reduction of ROS production and oxidative stress through the enhancement of Nrf2 expression. Moreover, PF extract rich in bioactive compounds such as polyphenols (caffeic acid, gallic acid, and flavonoids), β‐carotenoids, and γ‐tocopherols, as previously reported [27], may exert an antioxidant effect by enhancing antioxidant enzymes to counteract oxidative stress [52, 53]. Protein expression was evaluated in this study utilizing the western blot technique with 3*n*, which is an established minimum standard [54]. Additionally, inflammation occurs because of ROS–stimulated transcription factors and pro‐inflammatory genes. This results in oxidative stress and the reverse sequence of events, notably inflammation [55]. As previously stated, HFD enhances ROS, which stimulates the release of inflammatory cytokines [56]. In the same vein, our study observed that rats given an HFD exhibited high levels of two cytokines, IL‐6 and TNF‐α, via activating NF‐κB expression. Conversely, the PF extract and simvastatin groups showed a decrease in both cytokines. Additionally, prior studies have suggested that passion fruit has the ability to lower inflammatory cytokines that may be caused by elevated levels of Nrf2, which protect against HFD–induced oxidative stress and inflammation [57]. This suggests that PF extract and simvastatin may enhance Nrf2–mediated antioxidant properties, leading to a reduction in ROS–induced inflammation in skeletal muscle. CK and LDH are crucial enzymes that generate and utilize energy for muscular activity [58, 59]. In addition, these two enzymes are indicators for muscle injury [60]. The study found that the HFD group had higher CK and LDH levels in skeletal muscle than the control group. In comparison, a previous study reported that the HFD‐fed rats exhibited elevated CK and LDH levels [61]. Furthermore, elevated LDH activates inflammatory markers, resulting in the formation of necrotic factors that contribute to tissue injury [61]. Interestingly, PF extract and simvastatin were found to reduce CK and LDH levels in rats fed an HFD. Furthermore, our previous study reported that PF extract and simvastatin significantly reduced the levels of liver damage indicators, such as aspartate transaminase (AST) and ALT, when compared to the HFD group [25]. Previous reports have demonstrated that doses of passion fruit extract comparable to or higher than those used in the present study are safe and display hepatoprotective and nephroprotective properties [62, 63]. In addition, our current study assessed the toxicity of the extract and simvastatin based on our prior research, but standard indicators of renal damage, such as blood urea nitrogen and creatinine, were omitted, which is a limitation of this present study.

Skeletal muscle maintains glucose homeostasis by absorbing approximately 80% of glucose through insulin‐dependent glucose uptake. In this study, we investigated the effect of HFD on the *Slc2a4*, which is encoded by the glucose transporter‐4 (GLUT4) protein and is crucial for muscle glucose absorption that is regulated by hormones like insulin [64]. Insulin activates the IRS‐1/PI3K axis, leading to the translocation of GLUT4, which facilitates glucose absorption into muscle cells. Previous research has indicated that HFD can lead to a significant decrease in glucose absorption in skeletal muscle, ranging from 29% to 61%, and can induce IR [65]. Similarly, this study found that HFD decreased IRS‐1, *Pi3k*, and *Slc2a4* expressions, which reduced muscle glucose uptake while increasing IR determined by HOMA‐IR in obese rats. The PF extract increased IRS‐1/PI3K/AKT signaling pathway and *Slc2a4* expression. Although PF extract outperformed simvastatin in activating the IRS‐1/PI3K/AKT and *Slc2a4*, there was no difference in HOMA‐IR or glucose uptake. As previously reported, simvastatin may have a negative effect on glucose absorption in skeletal muscle due to decreased levels of proteins involved in glucose metabolism and IRS/AKT signaling [66]. However, both the PF extract and simvastatin indicated that they can improve glucose absorption and IR in HFD‐fed rats. This suggests that PF extract may prevent IR in skeletal muscle, partially from enhanced insulin sensitivity via the IRS‐1/PI3K/AKT signaling pathway and from increased glucose absorption via increasing *Slc2a4* expression. Similarly, a previous study showed that *P. edulis* peel flour improved insulin sensitivity in metabolic alterations produced by the cafeteria diet in mice [67]. Furthermore, glycogen is crucial in facilitating mobility within muscle tissues and serves as the primary site for insulin‐mediated glucose uptake. When energy is required by muscle cells, glycogen phosphorylase (GP) breaks down glycogen into glucose‐1‐phosphate, which is then metabolized through glycolysis to generate ATP [68]. According to our findings, HFD decreased glycogen and ATP contents in the GAS muscle. Similarly, a previous study found that HFD reduced muscle glycogen synthesis by around 20% due to IR and poor muscle glucose absorption [69]. Glycogen is strongly associated with muscle function, and a deficiency in glycogen leads to decreased muscular performance and the development of fatigue. Interestingly, a high dose of passion fruit extract demonstrated an increase in glycogen content by enhancing glucose absorption through activating the IRS‐1/PI3K/AKT, as well as *Slc2a4* expression.

BCL2 and BAX play crucial roles in the formation of mitochondrial apoptosis [70]. The mitochondrial system regulates skeletal muscle mass and function, which can be disrupted by a variety of metabolic disorders, including diabetes, obesity, and cancer [71]. Previously, the ratio of pro‐apoptotic BAX and anti‐apoptotic BCL2 levels in muscle were investigated, and both were reported to be elevated in the skeletal muscle of HFD‐fed mice [72]. Our findings show that HFD raises *Bax* gene expression while reducing *Bcl2* gene expression, resulting in an increase in the *Bax/Bcl2* ratio in HFD‐fed rats. In contrast, PF extract and simvastatin reduced *Bax*, whereas only 500 mg/kg of PF extract enhanced *Bcl2* expression. The balance of protein synthesis and breakdown rates regulates the size and mass of skeletal muscles [73]. TRIM36, which functions as an E3 ubiquitin ligase, is specifically present in skeletal and cardiac muscles. FBXO32 demonstrates significantly heightened levels during periods of muscle breakdown. Both TRIM36 and FBXO32 play crucial roles in the development of muscle atrophy. Meanwhile, MTOR, which interacts with both skeletal muscle hypertrophy and atrophy factors, is one of the most well‐known key players in muscle mass regulation [74]. Our findings show that the HFD enhances *Trim36* and *Fbxo32* expressions while reducing p‐AKT and *Mtor*. As a result, the HFD group had a reduction in CSA and muscle mass by about 10%–35%. Similarly, previous studies showed that HFD reduced muscle weight by about 10%–20% and CSA via upregulating TRIM36 and FBXO32 expressions [75]. Nevertheless, it has been observed that PF extract has the potential to improve CSA and muscle weight by upregulating the PI3K/p‐AKT/MTOR signaling pathway and downregulating *Trim36* and *Fbxo32*. In a similar vein, quercetin, a polyphenolic flavonoid found in passion fruit, has been shown to safeguard against obesity‐induced skeletal muscle atrophy by promoting muscle weight and fiber size while decreasing the expression of TRIM36 and FBXO32 [76]. The result suggests that PF extract prevents muscle atrophy via activating the PI3K/p‐AKT/MTOR signaling pathway while also inhibiting *Trim36* and *Fbxo32* expressions in the muscle of obese rats induced by an HFD. This finding implies that PF extract protects against muscle loss by modifying protein turnover via modulation of the IRS‐1/PI3K/AKT signaling, as well as suppressing genes related to muscle degradation and decreasing the *Bax/Bcl2* ratio in the muscle of obese rats.

As stated in the introduction, simvastatin is widely used as a lipid‐lowering drug, although it is associated with muscle‐damaging side effects. Interestingly, at a dose of 40 mg/kg BW, simvastatin exhibited a protective effect against muscular atrophy via suppressing genes involved with protein degradation, which contradicted the previous studies [18–20]. This may occur through simvastatin lowering lipid levels and limiting lipid accumulation in muscle tissue, thereby decreasing lipotoxicity and subsequent muscle atrophy. In addition, the contradiction may be due to the dosage of the drug and the duration of usage [18, 77]. The safety of the simvastatin dose used at 40 mg/kg is supported by our findings, which showed no adverse effects on the muscles under these experimental conditions; nevertheless, simvastatin should be administered with caution and in appropriate doses for a limited duration. Moreover, passion fruit extract exerted a comparable effect against myosteatosis to simvastatin. Notably, the extract showed greater benefit in some parameters, including anti‐apoptosis and muscle atrophy prevention. These findings suggest a practical approach of using simvastatin or an alternative extract in reducing adverse effects on muscle in obese. The extract may be considered as an alternative approach to simvastatin under some conditions, that is, statin intolerance or contraindications to statin.

## 5. Conclusion

**Figure 10:**
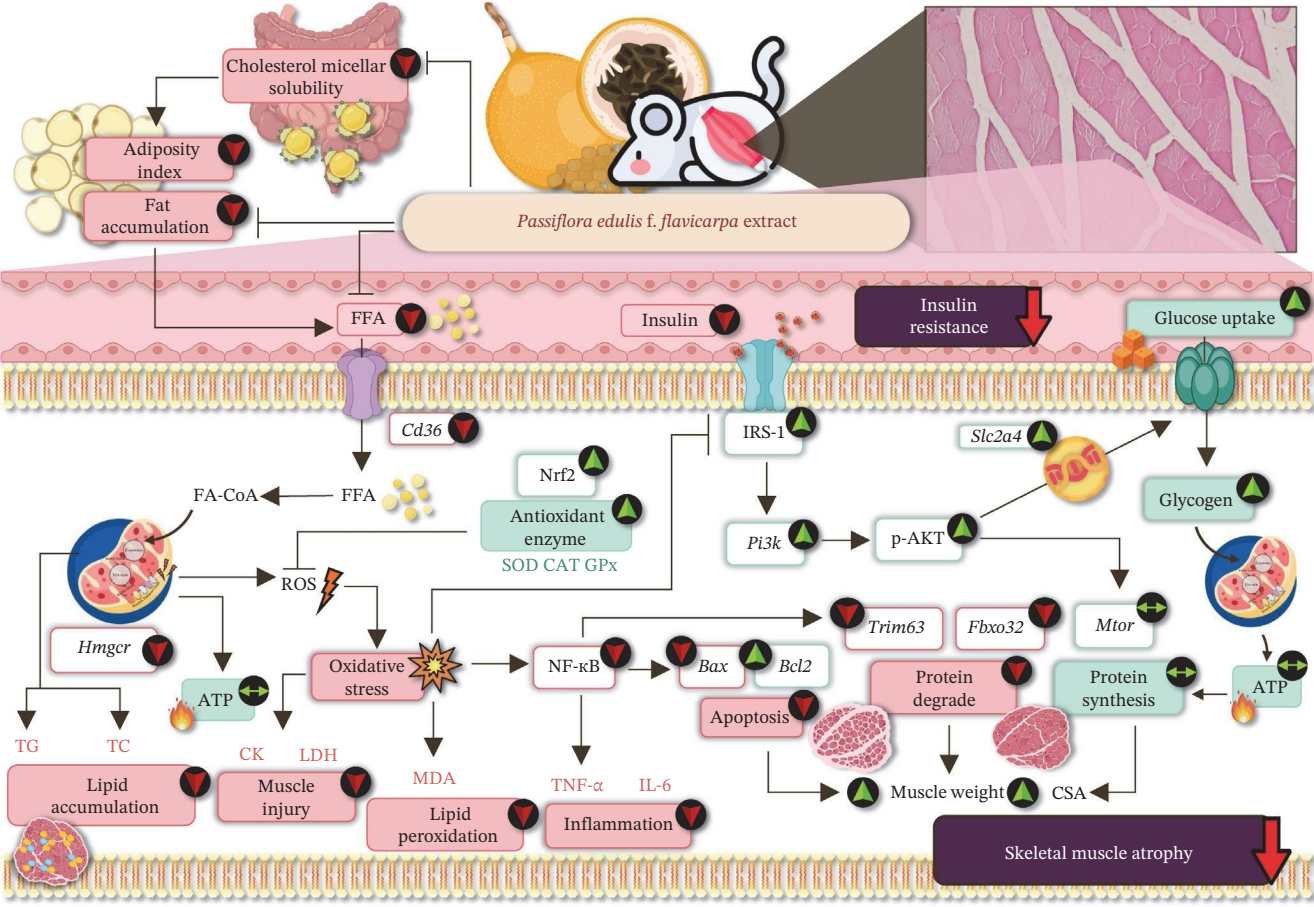
The potential mechanism of *Passiflora edulis* f. *flavicarpa* pulp extract in HFD–induced muscle atrophy and insulin‐resistant obesity in rats. PF extract reduced cholesterol micellar solubility, leading to decreased plasma FFAs levels and fat accumulation in adipose and muscle tissues. FFAs absorption into the myocyte was decreased, thereby mitigating adverse effects including oxidative stress, inflammation, muscle damage, insulin resistance, and muscle atrophy through the regulation of the IRS‐1/PI3K/AKT signaling pathway.

In conclusion, our findings demonstrate that PF extract has potential as a preventive agent for obesity and related metabolic diseases through reducing cholesterol micellar solubility and fat accumulation in adipose tissue and skeletal muscles of obese rats. Moreover, this study shows that PF extract protects against fat accumulation by inhibiting *Cd36* and *Hmgcr* gene expressions, as well as improving oxidative stress, muscle damage, and inflammation by increasing Nrf2 expression, and antioxidant enzymes, as well as decreasing NF‐κB expression. The findings also emphasize muscular atrophy and IR in obese rats. The effectiveness of PF extract was demonstrated in preventing muscle atrophy through enhancing protein synthesis by upregulating the PI3K/AKT signaling axis and *Bcl2* expressions while decreasing *Trim36*, *Fbxo32*, and *Bax* expressions. There was also IR prevention when the PF extract was administered in the obese rat model by upregulating the *Slc2a4* and IRS‐1/PI3K/AKT insulin signaling pathway. The schematic representation in Figure 10 illustrates the possible mechanism of PF in HFD‐induced obese rats. Thus, these findings suggest a potential alternative approach of PF extract, which works as effectively as simvastatin to prevent fat accumulation and oxidative stress‐associated inflammation, muscle damage, atrophy, and improved insulin sensitivity in the skeletal muscle of obese rats induced by an HFD. However, clinical studies are important. Therefore, future clinical trials should evaluate the ability of PF extract to mitigate IR and muscle atrophy.

**Nomenclature**
AKT: Protein kinase B
BAX: BCL2‐associated X
BCL2: B‐cell lymphoma 2
FBXO32: Muscle atrophy F‐box
HFD: High‐fat diets
HMGCR: 3‐Hydroxy‐3‐methylglutaryl coenzyme A reductase
IR: Insulin resistance
IRS‐1: Insulin receptor substrate‐1
MTOR: Mammalian target of rapamycin
NF‐κB: Nuclear factor kappa B
Nrf2: Nuclear factor erythroid 2‐related factor 2
PI3K: Phosphatidylinositol‐3 kinase
ROS: Reactive oxygen species
TRIM63: Muscle‐specific RING finger protein 1.

## Author Contributions

**Nrarat Chobsuay**: methodology, formal analysis, investigation, writing – original draft preparation, writing – review and editing. **Pennapa Chonpathompikunlert, Nanteetip Limpeanchob, and Sathid Aimjongjun**: conceptualization, methodology, resources. **Jukkarin Srivilai:** methodology, formal analysis of active compounds, writing – review. **Wachirawadee Malakul**: conceptualization, methodology, resources. **Sakara Tunsophon**: conceptualization, funding acquisition, supervision, analysis, writing – original draft preparation, writing – review and editing.

## Funding

This work was supported by Naresuan University (NU) and the National Science, Research and Innovation Fund (NSRF) (Grant R2565B048), the Global and Frontier Research University Fund, Naresuan University (Grant R2567C003), the Center of Excellence for Innovation in Chemistry (PERCH‐CIC), Ministry of Higher Education, Science, Research and Innovation, Thailand, and the Frontier Research and Innovation Cluster, Naresuan University (Grant R2569C004).

## Disclosure

The funders had no role in any parts of the research including study design, data collection and analysis, decision to publish, or preparation of the manuscript.

## Ethics Statement

Protocols for animal experiments were approved by the Naresuan University Center for Animal Research Ethics Committee (Ethics Approval Number NU‐AE 621023).

## Conflicts of Interest

The authors declare no conflicts of interest.

## Supporting Information

Additional supporting information can be found online in the Supporting Information section.

## Supporting information


**Supporting Information 1** Table S1. The primary antibodies used in this study. Table S2. Sequences of primers used for real‐time PCR in this study.

**Supporting Information 2** Figure S1 shows the calibration curves for gallic acid and caffeic acid utilized in this study.

## Data Availability

The data that support the findings of this study are available from the corresponding author upon reasonable request.

## References

[1] Jun L. , Robinson M. , Geetha T. , Broderick T. L. , and Babu J. R. , Prevalence and Mechanisms of Skeletal Muscle Atrophy in Metabolic Conditions, International Journal of Molecular Sciences. (2023) 24, no. 3, 10.3390/ijms24032973, 2973. 36769296 PMC9917738

[2] Valenzuela P. L. , Maffiuletti N. A. , Tringali G. , De Col A. , and Sartorio A. , Obesity-Associated Poor Muscle Quality: Prevalence and Association With Age, Sex, and Body Mass Index, BMC Musculoskeletal Disorders. (2020) 21, no. 1, 10.1186/s12891-020-03228-y, 21. 32234006 PMC7110672

[3] Huang Y. , Zhu X. , and Chen K. , et al.Resveratrol Prevents Sarcopenic Obesity by Reversing Mitochondrial Dysfunction and Oxidative Stress via the PKA/LKB1/AMPK Pathway, Aging. (2019) 11, no. 8, 2217–2240, 10.18632/aging.101910, 2-s2.0-85065599429. 30988232 PMC6519996

[4] Da Ressurreição S. , Pedreiro Sónia , Batista M. T. , and Figueirinha A. , Effect of Phenolic Compounds from Cymbopogon citratus (DC) Stapf. Leaves on Micellar Solubility of Cholesterol, Molecules. (2022) 27, no. 21, 10.3390/molecules27217338, 7338. 36364172 PMC9655935

[5] de Sousa L. G. O. , Marshall A. G. , and Norman J. E. , et al.The Effects of Diet Composition and Chronic Obesity on Muscle Growth and Function, Journal of Applied Physiology. (2021) 130, no. 1, 124–138, 10.1152/japplphysiol.00156.2020. 33211595 PMC7944928

[6] Chand S. , Tripathi A. S. , and Hasan T. , et al.Geraniol Reverses Obesity by Improving Conversion of Wat to Bat in High Fat Diet Induced Obese Rats by Inhibiting HMGCoA Reductase, Nutrition & Diabetes. (2023) 13, no. 1, 10.1038/s41387-023-00254-2. PMC10698077 38052812

[7] Seifert E. L. , Estey C. , Xuan J. Y. , and Harper M.-E. , Electron Transport Chain-Dependent and -Independent Mechanisms of Mitochondrial H_2_O_2_ Emission During Long-Chain Fatty Acid Oxidation, Journal of Biological Chemistry. (2010) 285, no. 8, 5748–5758, 10.1074/jbc.M109.026203, 2-s2.0-77949322975. 20032466 PMC2820802

[8] Tumova J. , Andel M. , and Trnka J. , Excess of Free Fatty Acids as a Cause of Metabolic Dysfunction in Skeletal Muscle, Physiological Research. (2016) 65, 193–207, 10.33549/physiolres.932993. 26447514

[9] Jena A. B. , Samal R. R. , Bhol N. K. , and Duttaroy A. K. , Cellular Red-Ox System in Health and Disease: The Latest Update, Biomedicine & Pharmacotherapy. (2023) 162, 10.1016/j.biopha.2023.114606, 114606. 36989716

[10] Talib W. H. , AL-ataby I. A. , Mahmod A. I. , Jawarneh S. , Al Kury L. T. , and AL-Yasari I. H. , The Impact of Herbal Infusion Consumption on Oxidative Stress and Cancer: The Good, the Bad, the Misunderstood, Molecules. (2020) 25, no. 18, 10.3390/molecules25184207, 4207. 32937891 PMC7570648

[11] Sebe M. , Tsutsumi R. , and Senoura S. , et al.Saturated Fatty Acids Intake is Associated With Muscle Atrophy in Rheumatoid Arthritis, JCSM Rapid Communications. (2022) 5, no. 1, 86–101, 10.1002/rco2.53.

[12] Collino M. , Mastrocola R. , and Nigro D. , et al.Variability in Myosteatosis and Insulin Resistance Induced by High-Fat Diet in Mouse Skeletal Muscles, BioMed Research International. (2014) 2014, 1–10, 10.1155/2014/569623, 2-s2.0-84907411169. PMC4147206 25197650

[13] Shen Y. , Li M. , and Wang K. , et al.Diabetic Muscular Atrophy: Molecular Mechanisms and Promising Therapies, Frontiers in Endocrinology. (2022) 13, 10.3389/fendo.2022.917113, 13. PMC9279556 35846289

[14] Wu Y. , Yanhui Y. , and Caixia D. , et al.Berberine Attenuates Obesity-Induced Skeletal Muscle Atrophy via Regulation of FUNDC1 in Skeletal Muscle of Mice, Scientific Reports. (2025) 15, no. 1, 10.1038/s41598-025-89297-2. PMC11811154 39930016

[15] Funai K. , Song H. , and Yin L. , et al.Muscle Lipogenesis Balances Insulin Sensitivity and Strength Through Calcium Signaling, Journal of Clinical Investigation. (2013) 123, no. 3, 1229–1240, 10.1172/JCI65726, 2-s2.0-84874624368. 23376793 PMC3582136

[16] Eslamparast T. , Montano-Loza A. J. , Raman M. , and Tandon P. , Sarcopenic Obesity in Cirrhosis—the Confluence of 2 prognostic Titans, Liver International. (2018) 38, no. 10, 1706–1717, 10.1111/liv.13876, 2-s2.0-85053879311. 29738109

[17] LiverTox: Clinical and Research Information on Drug-Induced Liver Injury, 2012, National Institute of Diabetes and Digestive and Kidney Diseases. 31643176

[18] Goodman C. A. , Pol D. , and Zacharewicz E. , et al.Statin-Induced Increases in Atrophy Gene Expression Occur Independently of Changes in PGC1α Protein and Mitochondrial Content, PLOS ONE. (2015) 10, no. 5, 10.1371/journal.pone.0128398, 2-s2.0-84933055205. PMC4447258 26020641

[19] Arora R. , Liebo M. , and Maldonado F. , Statin-Induced Myopathy: The Two Faces of Janus, Journal of Cardiovascular Pharmacology and Therapeutics. (2006) 11, no. 2, 105–112, 10.1177/1074248406288758, 2-s2.0-33748995423. 16891287

[20] Parkin L. , Paul C. , and Herbison G. P. , Simvastatin Dose and Risk of Rhabdomyolysis: Nested Case–Control Study Based on National Health and Drug Dispensing Data, International Journal of Cardiology. (2014) 174, no. 1, 83–89, 10.1016/j.ijcard.2014.03.150, 2-s2.0-84901194723. 24726164

[21] He X. , Luan F. , and Yang Y. , et al. *Passiflora Edulis*: An Insight Into Current Researches on Phytochemistry and Pharmacology, Frontiers in Pharmacology. (2020) 11, 10.3389/fphar.2020.00617, 11. 32508631 PMC7251050

[22] dos Reis L. C. R. , Facco E. M. P. , Salvador M. , Flôres S. H. , and de Oliveira Rios A. , Antioxidant Potential and Physicochemical Characterization of Yellow, Purple and Orange Passion Fruit, Journal of Food Science and Technology. (2018) 55, no. 7, 2679–2691, 10.1007/s13197-018-3190-2, 2-s2.0-85046491220. 30042584 PMC6033812

[23] Pereira Z. C. , dos Anjos Cruz J. M. , and Corrêa R. F. , et al.Passion Fruit (*Passiflora* spp.) Pulp: A Review on Bioactive Properties, Health Benefits and Technological Potential, Food Research International. (2023) 166, 10.1016/j.foodres.2023.112626, 112626. 36914332

[24] Adi D. I. , The Effect Yellow Passion Fruit Peel Juice (*Passiflora Eduils f. Flavicarpa Deg*) on LDL to HDL Cholesterol Ratio in Type 2 Diabetes Mellitus Patients as Predictors of Cardiovascular Disease, Jurnal Gizi Dan Dietetik Indonesia (Indonesian Journal of Nutrition and Dietetics). (2021) 8, no. 2, 10.21927/ijnd.2020.8(2).61-67, 61.

[25] Kinge A. , Wadatkar J. , and Sakhare D. , A Review: Pharmacological and Phytochemical Update of Passiflora edulis F. Flavicarpa, GSC Biological and Pharmaceutical Sciences. (2024) 27, no. 2, 143–153, 10.30574/gscbps.2024.27.2.0177.

[26] Jako P. , Chonpathompikunlert P. , Malakul W. , and Tunsophon S. , Extract Ameliorates HFD-Induced Hepatic Steatosis Mediated Through NRF2 and IRS-1 Activation, NFKB Suppression, and Hepatic Lipid Metabolism and Bile Acid Modulation in Obese Rats, Journal of Functional Foods. (2024) 120, 10.1016/j.jff.2024.106351, 106351.

[27] Sukketsiri W. , Daodee S. , and Parhira S. , et al.Chemical Characterization of, *Passiflora Edulis*, Extracts and Their, *In Vitro*, Antioxidant, Anti-Inflammatory, Anti-Lipid Activities, and, *Ex-Vivo*, Vasodilation Effect, Journal of King Saud University - Science. (2023) 35, no. 1, 10.1016/j.jksus.2022.102431, 102431.

[28] Kandandapani S. , Balaraman A. K. , and Ahamed H. N. , Extracts of Passion Fruit Peel and Seed of *Passiflora Edulis* (Passifloraceae) Attenuate Oxidative Stress in Diabetic Rats, Chinese Journal of Natural Medicines. (2015) 13, no. 9, 680–686, 10.1016/S1875-5364(15)30066-2, 2-s2.0-84942123448. 26412428

[29] Duangjai A. , Limpeanchob N. , Trisat K. , and Amornlerdpison D. , Spirogyra Neglecta Inhibits the Absorption and Synthesis of Cholesterol In Vitro, Integrative Medicine Research. (2016) 5, no. 4, 301–308, 10.1016/j.imr.2016.08.004. 28462132 PMC5390754

[30] Mu R.-F. , Niu Y.-F. , and Wang Q. , et al.Eriocalyxin B Inhibits Adipogenesis in 3T3-L1 Adipocytes by Cell Cycle Arrest, Natural Products and Bioprospecting. (2020) 10, no. 3, 131–140, 10.1007/s13659-020-00240-6. 32314168 PMC7253553

[31] Reddy M. S. K. and Manjappara U. V. , Capsaicin and Genistein Override the Action of Obestatin to Decrease Lipid Accumulation in 3T3-L1 Cells, Cell Biochemistry and Biophysics. (2019) 77, no. 3, 245–252, 10.1007/s12013-019-00875-4, 2-s2.0-85072153017. 31134453

[32] du Sert N. Percie , Hurst V. , and Ahluwalia A. , et al.The ARRIVE Guidelines 2.0: Updated Guidelines for Reporting Animal Research, BMC Veterinary Research. (2020) 16, no. 1, 10.1186/s12917-020-02451-y. PMC7359286 32660541

[33] Janson B. , Prasomthong J. , Malakul W. , Boonsong T. , and Tunsophon S. , Hibiscus Sabdariffa *L. calyx* Extract Prevents the Adipogenesis of 3T3-L1 Adipocytes, and Obesity-Related Insulin Resistance in High-Fat Diet-Induced Obese Rats, Biomedicine & Pharmacotherapy. (2021) 138, 10.1016/j.biopha.2021.111438, 111438. 33721756

[34] Leopoldo A. S. , Lima-Leopoldo A. P. , and Nascimento A. F. , et al.Classification of Different Degrees of Adiposity in Sedentary Rats, Brazilian Journal of Medical and Biological Research. (2016) 49, no. 4, 10.1590/1414-431X20155028, 2-s2.0-84975709152. PMC4792506 26909787

[35] Bowyer D. E. , Cridland J. S. , and King J. P. , A Novel Semiautomated Method for the Estimation of Free Fatty Acid in Serum or Plasma, Journal of Lipid Research. (1978) 19, no. 2, 274–280, 10.1016/S0022-2275(20)41568-8. 632690

[36] Widyawati T. , Yusoff N. , Asmawi M. , and Ahmad M. , Antihyperglycemic Effect of Methanol Extract of *Syzygium Polyanthum* (Wight.) Leaf in Streptozotocin-Induced Diabetic Rats, Nutrients. (2015) 7, no. 9, 7764–7780, 10.3390/nu7095365, 2-s2.0-84941695344. 26389944 PMC4586560

[37] Huang M. , Wang P. , and Xu S. , et al.Biological Activities of Salvianolic Acid B From *Salvia Miltiorrhiza* on Type 2 Diabetes Induced by High-Fat Diet and Streptozotocin, Pharmaceutical Biology. (2015) 53, no. 7, 1058–1065, 10.3109/13880209.2014.959611, 2-s2.0-84930195047. 25612777

[38] Bastías-Pérez M. , Serra D. , and Herrero L. , Dietary Options for Rodents in the Study of Obesity, Nutrients. (2020) 12, no. 11, 10.3390/nu12113234, 3234. 33105762 PMC7690621

[39] Marques C. , Meireles M. , and Norberto S. , et al.High-Fat Diet-Induced Obesity Rat Model: A Comparison Between Wistar and Sprague-Dawley Rat, Adipocyte. (2015) 5, no. 1, 11–21, 10.1080/21623945.2015.1061723, 2-s2.0-85063828922. 27144092 PMC4836488

[40] Hariri N. and Thibault L. , High-Fat Diet-Induced Obesity in Animal Models, Nutrition Research Reviews. (2010) 23, no. 2, 270–299, 10.1017/S0954422410000168, 2-s2.0-78650535334. 20977819

[41] Melo A. B. , Damiani A. P. L. , and Coelho P. M. , et al.Resistance Training Promotes Reduction in Visceral Adiposity Without Improvements in Cardiomyocyte Contractility and Calcium Handling in Obese Rats, International Journal of Medical Sciences. (2020) 17, no. 12, 1819–1832, 10.7150/ijms.42612. 32714085 PMC7378665

[42] Vuolo M. M. , Lima G. C. , and Batista Â.G. , et al.Passion Fruit Peel Intake Decreases Inflammatory Response and Reverts Lipid Peroxidation and Adiposity in Diet-Induced Obese Rats, Nutrition Research. (2020) 76, 106–117, 10.1016/j.nutres.2019.08.007. 32033839

[43] Holloway G. P. , Chou C. J. , and Lally J. , et al.Increasing Skeletal Muscle Fatty Acid Transport Protein 1 (FATP1) Targets Fatty Acids to Oxidation and Does Not Predispose Mice to Diet-Induced Insulin Resistance, Diabetologia. (2011) 54, no. 6, 1457–1467, 10.1007/s00125-011-2114-8, 2-s2.0-80052538942. 21442160

[44] Yang J. S. , Tongson J. , Kim K.-H. , and Park Y. , Piceatannol Attenuates Fat Accumulation and Oxidative Stress in Steatosis-Induced HepG2 Cells, Current Research in Food Science. (2020) 3, 92–99, 10.1016/j.crfs.2020.03.008. 32914125 PMC7473378

[45] Jia H. , Tian L. , Zhang B. , Fan X. , and Zhao D. , The Soluble Fraction of Soy Protein Peptic Hydrolysate Reduces Cholesterol Micellar Solubility and Uptake, International Journal of Food Science & Technology. (2019) 54, no. 6, 2123–2131, 10.1111/ijfs.14117, 2-s2.0-85060971620.

[46] Thilavech T. and Adisakwattana S. , Cyanidin-3-Rutinoside Acts as a Natural Inhibitor of Intestinal Lipid Digestion and Absorption, BMC Complementary and Alternative Medicine. (2019) 19, no. 1, 10.1186/s12906-019-2664-8, 2-s2.0-85071747946. PMC6727418 31488210

[47] Trisat K. and Limpeanchob N. , Impact of, *Artocarpus Lakoocha*, Heartwood Extract and Oxyresveratrol on Cholesterol Digestion and Absorption in Caco-2 Cell Cultures, Journal of Applied Pharmaceutical Science. (2025) 15, 65–71, 10.7324/JAPS.2025.218916.

[48] Meex R. C. R. , Blaak E. E. , and van Loon L. J. C. , Lipotoxicity Plays a Key Role in the Development of Both Insulin Resistance and Muscle Atrophy in Patients With Type 2 Diabetes, Obesity Reviews. (2019) 20, no. 9, 1205–1217, 10.1111/obr.12862, 2-s2.0-85068313733. 31240819 PMC6852205

[49] Cordiano R. , Di Gioacchino M. , Mangifesta R. , Panzera C. , Gangemi S. , and Minciullo P. L. , Malondialdehyde as a Potential Oxidative Stress Marker for Allergy-Oriented Diseases: An Update, Molecules. (2023) 28, no. 16, 10.3390/molecules28165979, 5979. 37630231 PMC10457993

[50] Wei Y. , Zhang J. , and Yan X. , et al.Remarkable Protective Effects of Nrf2-Mediated Antioxidant Enzymes and Tissue Specificity in Different Skeletal Muscles of Daurian Ground Squirrels Over the Torpor-Arousal Cycle, Frontiers in Physiology. (2019) 10, 10.3389/fphys.2019.01449, 10. 31824343 PMC6883408

[51] He H.-J. , Curcumin Attenuates Nrf2 Signaling Defect, Oxidative Stress in Muscle and Glucose Intolerance in High Fat Diet-Fed Mice, World Journal of Diabetes. (2012) 3, no. 5, 10.4239/wjd.v3.i5.94, 94. 22645638 PMC3360224

[52] Althurwi H. N. , Abdel-Rahman R. F. , and Soliman G. A. , et al.Protective Effect of *Beta*-Carotene Against Myeloperoxidase- Mediated Oxidative Stress and Inflammation in Rat Ischemic Brain Injury, Antioxidants. (2022) 11, no. 12, 10.3390/antiox11122344, 2344. 36552554 PMC9774247

[53] Sanjay S. , Girish C. , Toi P. C. , and Bobby Z. , Gallic Acid Attenuates Isoniazid and Rifampicin-Induced Liver Injury by Improving Hepatic Redox Homeostasis Through Influence on Nrf2 and NF-κB Signalling Cascades in Wistar Rats, Journal of Pharmacy and Pharmacology. (2021) 73, no. 4, 473–486, 10.1093/jpp/rgaa048. 33793834

[54] Wu L. , Hu X. , Tang H. , Han Z. , and Chen Y. , Valid Application of Western Blotting, Molecular Biology Reports. (2014) 41, no. 5, 3517–3520, 10.1007/s11033-014-3215-5, 2-s2.0-84905703030. 24510387

[55] Jiang S. , Liu H. , and Li C. , Dietary Regulation of Oxidative Stress in Chronic Metabolic Diseases, Foods. (2021) 10, no. 8, 10.3390/foods10081854, 1854. 34441631 PMC8391153

[56] Baird M. F. , Graham S. M. , Baker J. S. , and Bickerstaff G. F. , Creatine-Kinase- and Exercise-Related Muscle Damage Implications for Muscle Performance and Recovery, Journal of Nutrition and Metabolism. (2012) 2012, 1–13, 10.1155/2012/960363, 2-s2.0-84866482920. PMC3263635 22288008

[57] Bartoloni B. , Mannelli M. , Gamberi T. , and Fiaschi T. , The Multiple Roles of Lactate in the Skeletal Muscle, Cells. (2024) 13, no. 14, 10.3390/cells13141177, 1177. 39056759 PMC11274880

[58] Andrich D. E. , Melbouci L. , and Ou Y. , et al.A Short-Term High-Fat Diet Alters Glutathione Levels and IL-6 Gene Expression in Oxidative Skeletal Muscles of Young Rats, Frontiers in Physiology. (2019) 10, 10.3389/fphys.2019.00372, 2-s2.0-85068215812. PMC6468044 31024337

[59] Gao W. , Guo L. , and Yang Y. , et al.Dissecting the Crosstalk Between Nrf2 and NF-KB Response Pathways in Drug-Induced Toxicity, Frontiers in Cell and Developmental Biology. (2022) 9, 10.3389/fcell.2021.809952. PMC8847224 35186957

[60] Rodrigues P. , Wassmansdorf R. , and Salgueirosa F. M. , et al.Time-Course of Changes in Indirect Markers of Muscle Damage Responses Following a 130-Km Cycling Race, Brazilian Journal of Kinanthropometry and Human Performance. (2016) 18, no. 3, 10.5007/1980-0037.2016v18n3p322, 2-s2.0-84978733907, 322.

[61] Feriani A. , Bizzarri M. , and Tir M. , et al.High-Fat Diet-Induced Aggravation of Cardiovascular Impairment in Permethrin-Treated Wistar Rats, Ecotoxicology and Environmental Safety. (2021) 222, 10.1016/j.ecoenv.2021.112461, 112461. 34224971

[62] Konta E. M. , Almeida M. R. , and Amaral Cátia Ldo , et al.Evaluation of the Antihypertensive Properties of Yellow Passion Fruit Pulp (*Passiflora edulis* Sims f. *Flavicarpa*, Deg.) in Spontaneously Hypertensive Rats, Phytotherapy Research. (2014) 28, no. 1, 28–32, 10.1002/ptr.4949, 2-s2.0-84892432432. 23436457

[63] Nerdy N. and Ritarwan K. , Hepatoprotective Activity and Nephroprotective Activity of Peel Extract From Three Varieties of the Passion Fruit (*Passiflora Sp*.) in the Albino Rat, Open Access Macedonian Journal of Medical Sciences. (2019) 7, no. 4, 536–542, 10.3889/oamjms.2019.153, 2-s2.0-85064134752. 30894908 PMC6420947

[64] Jha D. and Mitra Mazumder P. , High Fat Diet Administration Leads to the Mitochondrial Dysfunction and Selectively Alters the Expression of Class 1 GLUT Protein in Mice, Molecular Biology Reports. (2019) 46, no. 2, 1727–1736, 10.1007/s11033-019-04623-y, 2-s2.0-85061215850. 30725350

[65] Pataky M. W. , Wang H. , and Yu C. S. , et al.High-Fat Diet-Induced Insulin Resistance in Single Skeletal Muscle Fibers is Fiber Type Selective, Scientific Reports. (2017) 7, no. 1, 10.1038/s41598-017-12682-z, 2-s2.0-85032012399. PMC5651812 29057943

[66] Galicia-Garcia U. , Jebari S. , and Larrea-Sebal A. , et al.Statin Treatment-Induced Development of Type 2 Diabetes: From Clinical Evidence to Mechanistic Insights, International Journal of Molecular Sciences. (2020) 21, no. 13, 10.3390/ijms21134725, 4725. 32630698 PMC7369709

[67] Goss M. J. , Nunes M. L. O. , and Machado I. D. , et al.Flavicarpa Supplementation Prevents the Insulin Resistance and Hepatic Steatosis Induced by Low-Fructose-Diet in Young Rats, Biomedicine & Pharmacotherapy. (2018) 102, 848–854, 10.1016/j.biopha.2018.03.137, 2-s2.0-85044577685. 29605773

[68] Ørtenblad N. , Westerblad H. , and Nielsen J. , Muscle Glycogen Stores and Fatigue, The Journal of Physiology. (2013) 591, no. 18, 4405–4413, 10.1113/jphysiol.2013.251629, 2-s2.0-84884286967. 23652590 PMC3784189

[69] He J. and Kelley D. E. , Muscle Glycogen Content in Type 2 Diabetes Mellitus, American Journal of Physiology-Endocrinology and Metabolism. (2004) 287, no. 5, E1002–E1007, 10.1152/ajpendo.00015.2004, 2-s2.0-6044264975. 15251866

[70] Kashani Vahid N. , Nameni F. , and Yazdanparast Chaharmahali B. , Effect of Interval Training and Curcumin on BAX, Bcl-2, and Caspase-3 Enzyme Activity in Rats, Gene, Cell and Tissue. (2022) 9, no. 4, 10.5812/gct-112792.

[71] Romanello V. and Sandri M. , The Connection Between the Dynamic Remodeling of the Mitochondrial Network and the Regulation of Muscle Mass, Cellular and Molecular Life Sciences. (2021) 78, no. 4, 1305–1328, 10.1007/s00018-020-03662-0. 33078210 PMC7904552

[72] Abrigo J. , Rivera J. C. , and Aravena J. , et al.High Fat Diet-Induced Skeletal Muscle Wasting is Decreased by Mesenchymal Stem Cells Administration: Implications on Oxidative Stress, Ubiquitin Proteasome Pathway Activation, and Myonuclear Apoptosis, Oxidative Medicine and Cellular Longevity. (2016) 2016, no. 1, 10.1155/2016/9047821, 2-s2.0-84984679700. PMC4992759 27579157

[73] Steinert N. D. , Jorgenson K. W. , Lin K.-H. , Hermanson J. B. , Lemens J. L. , and Hornberger T. A. , A Novel Method for Visualizing in-Vivo Rates of Protein Degradation Provides Insight Into How TRIM28 Regulates Muscle Size, iScience. (2023) 26, no. 4, 10.1016/j.isci.2023.106526, 106526. 37070069 PMC10105291

[74] Yoon M.-S. , MTOR as a Key Regulator in Maintaining Skeletal Muscle Mass, Frontiers in Physiology. (2017) 8, 10.3389/fphys.2017.00788, 2-s2.0-85031807112, 8. 29089899 PMC5650960

[75] Zhang J. , Zhuang P. , and Wang Y. , et al.Reversal of Muscle Atrophy by Zhimu-Huangbai Herb-Pair via AKT/mTOR/FOXO3 Signal Pathway in Streptozotocin-Induced Diabetic Mice, PLoS ONE. (2014) 9, no. 6, 10.1371/journal.pone.0100918, 2-s2.0-84903376087. PMC4072704 24968071

[76] Park J. , Miyakawa T. , Shiokawa A. , Nakajima-Adachi H. , Tanokura M. , and Hachimura S. , Quercetin Protects Against Obesity-Induced Skeletal Muscle Inflammation and Atrophy, Mediators of Inflammation. (2014) 2014, 1–9, 10.1155/2014/826987, 2-s2.0-84896934419. PMC4295595 25614714

[77] Termkwancharoen C. , Malakul W. , Phetrungnapha A. , and Tunsophon S. , Naringin Ameliorates Skeletal Muscle Atrophy and Improves Insulin Resistance in High-Fat-Diet-Induced Insulin Resistance in Obese Rats, Nutrients. (2022) 14, no. 19, 10.3390/nu14194120, 4120. 36235772 PMC9571698

