# Supplementary material for: Passiflora edulis f. flavicarpa Extract Prevents Muscle Atrophy and Insulin Resistance in High‐Fat Diet–Induced Obese Rats via Regulating the Nrf2, NF‐κB, and IRS‐1/PI3K/AKT Signaling Pathways
Source: Oxid Med Cell Longev. 2026 May 4;2026:5709962. doi: 10.1155/omcl/5709962 (PMC13139765; doi:10.1155/omcl/5709962)
Supplement: Supplementary file 1 — Supporting Information 1 Table S1. The primary antibodies used in this study. Table S2. Sequences of primers used for real‐time PCR in this study. [file OMCL-2026-5709962-s001.docx]

# Supplementary Table 1. The primary antibodies used in this study.

| Antibody | Dilution factor | Company |
| --- | --- | --- |
| AKT | 1:500 | Cell signal |
| β-actin | 1:2000 | Elabscience Biotechnology |
| IRS-1 | 1:500 | Elabscience Biotechnology |
| NF-κB | 1:500 | Cell signal |
| Nrf2 | 1:500 | Affinity Biosciences |
| p-AKT | 1:500 | Cell signal |

AKT, protein kinase B; IRS-1, insulin receptor substrate-1; NF-κB, nuclear factor kappa B; Nrf2, nuclear factor erythroid 2-related factor 2; p-AKT, phosphorylated AKT.

# Supplementary Table 2. Sequences of primers used for real-time PCR in this study.

| Gene | Forward (5’-3’) | Reverse (5’-3’) | Annealing temperature |
| --- | --- | --- | --- |
| *Fbxo32* | AGACCGGCTACTGTGGAAGAG | CCGTGCATGGATCAGTG | 60°C |
| *Bax* | ACACCTGAGCTGACCTTG | AGCCCATGATGGTTCTGATC | 52°C |
| *Bcl2* | GATGACTTCTCTCGTCGCTACC | ACCCCATCCCTGAAGAGTTCC | 64°C |
| *Cd36* | CCTGTGAGTTGGCAAGAAGC | AATGAGCCCACAGTTCCGAT | 55 °C |
| *Gapdh* | TGCACCACCAACTGCTTA | GGATGCAGGGATGATGTTC | 60°C |
| *Hmgcr* | TGCGTGTCCCTGGTCCTA | TTGGGTTACTGGGTTTGG | 50 °C |
| *Mtor* | CAGGACGAGCGAGTGAT | CGAGTTGGTGGACAGAGG | 58°C |
| *Trim63* | ACAACCTCTGCCGGAAGTGT | CCGCGGTTGGTCCAGTAG | 55°C |
| *Pi3k* | GCCTGCTCTGTAGTGGTAGATG | GGAGGTGTGTTGGTAATGTAGC | 52°C |
| *Slc2a4* | GCTTCTGTTGCCCTTCTGTC | TGGACGCTCTCTTTCCAACT | 60°C |

*Fbxo32*, muscle atrophy F-box; *Bax*, BCL2-associated X; *Bcl2*, B-cell lymphoma 2; *Cd36*, cluster of differentiation 36; *Gapdh*, glyceraldehyde-3-phosphate dehydrogenase; *Hmgcr*, 3-hydroxy-3-methyl glutaryl coenzyme A reductase; *Mtor*, mammalian target of rapamycin; *Trim63*, muscle-specific RING finger protein 1; *Pi3k*, phosphatidylinositol-3 kinase; *Slc2a4*, solute carrier family 2 member 4.
