# Supplementary figures and images for: Passiflora edulis f. flavicarpa Extract Prevents Muscle Atrophy and Insulin Resistance in High‐Fat Diet–Induced Obese Rats via Regulating the Nrf2, NF‐κB, and IRS‐1/PI3K/AKT Signaling Pathways
Source: Oxid Med Cell Longev. 2026 May 4;2026:5709962. doi: 10.1155/omcl/5709962 (PMC13139765; doi:10.1155/omcl/5709962)

Calibration curve of gallic acid

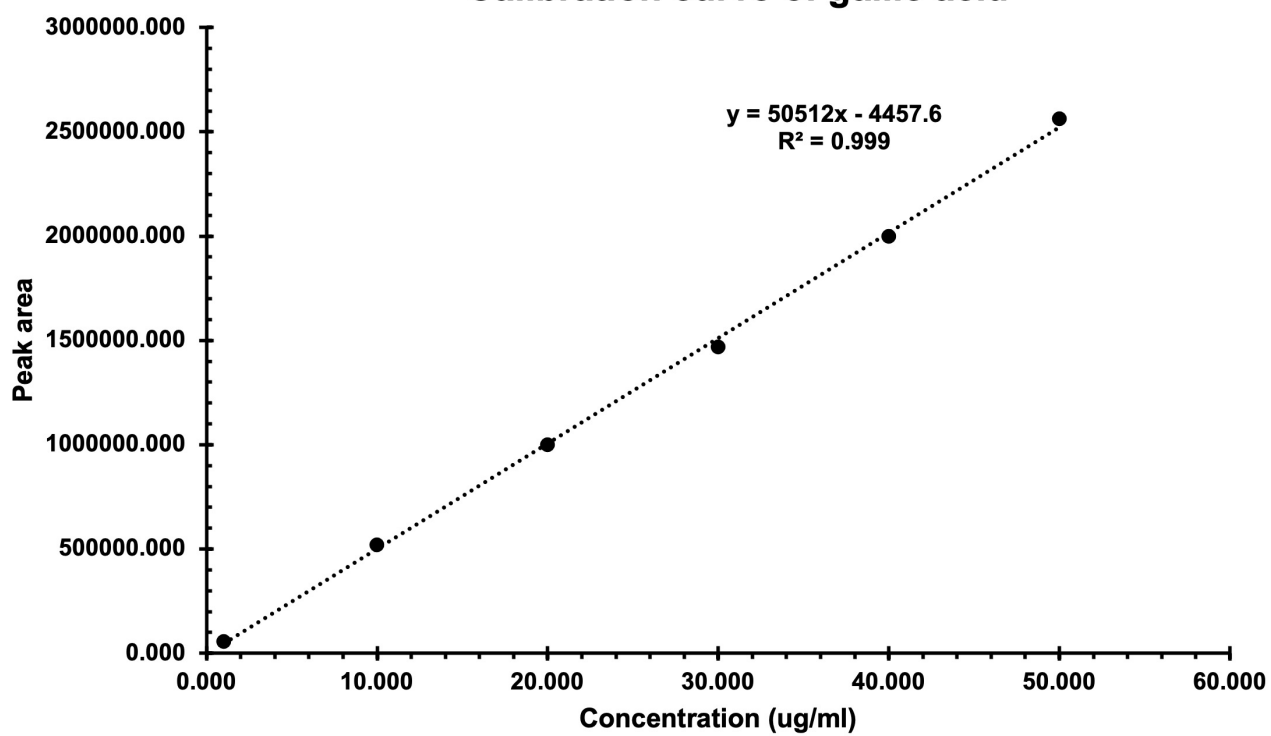

Calibration curve of caffeic acid

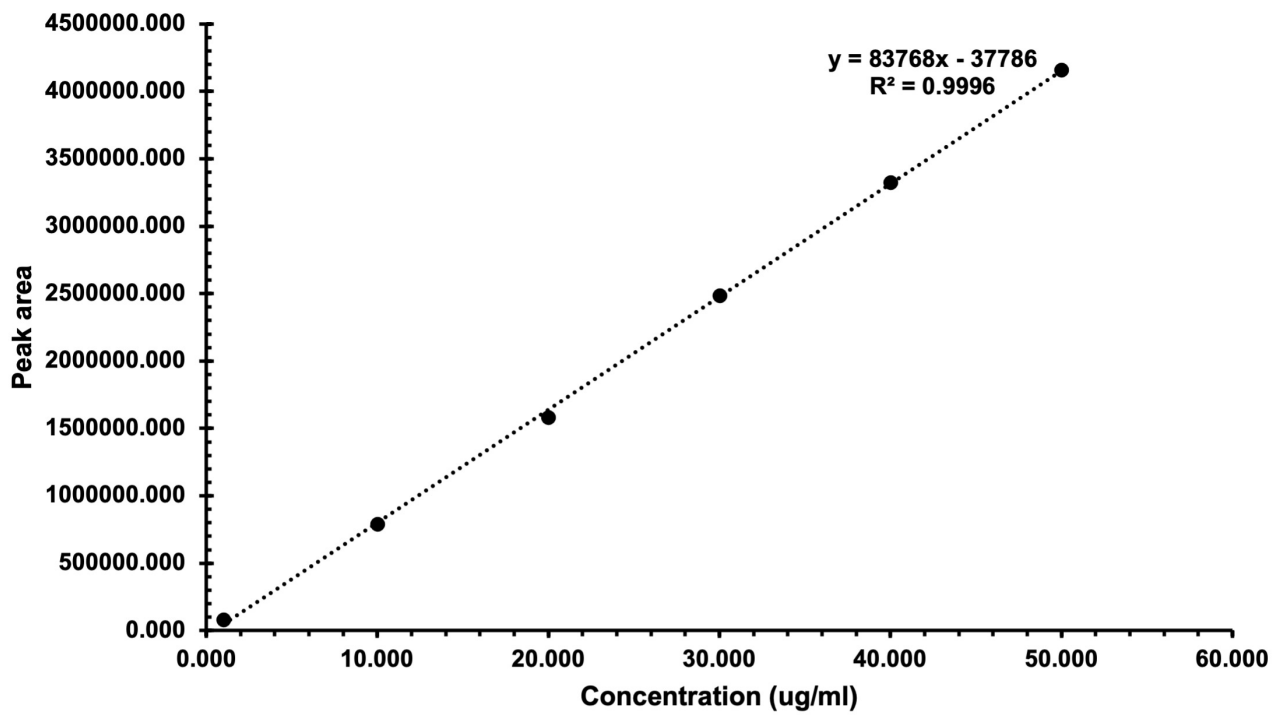

Supplement: Supplementary file 2 — Supporting Information 2 Figure S1 shows the calibration curves for gallic acid and caffeic acid utilized in this study. [file OMCL-2026-5709962-s002.pdf]
